# Evolution of *Listeria monocytogenes* During a Persistent Human Prosthetic Hip Joint Infection

**DOI:** 10.3389/fmicb.2020.01726

**Published:** 2020-07-28

**Authors:** Francis Muchaamba, Athmanya K. Eshwar, Ueli von Ah, Marc J. A. Stevens, Taurai Tasara

**Affiliations:** ^1^Institute for Food Safety and Hygiene, Vetsuisse Faculty, University of Zurich, Zurich, Switzerland; ^2^Agroscope, Bern, Switzerland

**Keywords:** *Listeria monocytogenes*, prosthetic joint infection, phenotype, genome, evolution

## Abstract

*Listeria monocytogenes* associated prosthetic joint infections (PJI) are a rare but increasing clinical problem of listeriosis. We characterized two isolates of the same *L. monocytogenes* strain isolated within five years of each other from a recurrent human prosthetic joint infection. The two isolates although clonally identical were phenotypically distinct confirming that the original infection strain had evolved within the human host PJI environment giving rise to a phenotypically distinct variant. The recurrent PJI isolate displayed various phenotypic differences compared to the parental original PJI isolate including diminished growth and carbon source metabolism, as well as altered morphology and increased stress sensitivity. The PJI isolates were both diminished in virulence due to an identical truncation mutation in the major virulence regulator PrfA. Genome wide sequence comparison provided conclusive evidence that the two isolates were identical clonal descendants of the same *L. monocytogenes* strain that had evolved through acquisition of various single nucleotide polymorphisms (SNPs) as well as insertion and deletion events (InDels) during a persistent human PJI. Acquired genetic changes included a specific mutation causing premature stop codon (PMSC) and truncation of RNAse J1 protein. Based on analysis of this naturally truncated as well as other complete RNAse J1 deletion mutants we show that the long-term survival of this specific *L. monocytogenes* strain within the prosthetic joint might in part be explained by the *rnjA* PMSC mutation that diminishes virulence and activation of the host immune system in a zebrafish embryo localized infection model. Overall our analysis of this special natural case provides insights into random mutation events and molecular mechanisms that might be associated with the adaptation and short-term evolution of this specific *L. monocytogenes* strain within a persistent human PJI environment.

## Introduction

Listeriosis is a serious foodborne disease caused by *Listeria monocytogenes*, predominantly affecting people with weakened immune systems including neonates, pregnant women, elderly and immunocompromised individuals leading to serious illness with high mortality rates (European Food Safety Authority [EFSA], 2017; Centers for Disease Control [CDC], 2018; Radoshevich and Cossart, 2018). Although infections usually manifest as meningitis, bacteremia, and feto-maternal complications, listeriosis can in rare cases present as focal infections in different organs including bone and joint (Allerberger and Wagner, 2010; Del Pozo et al., 2013; Chavada et al., 2014; European Food Safety Authority [EFSA], 2017; Radoshevich and Cossart, 2018). Septic arthritis is one of infrequent listeriosis manifestation that usually involves orthopedic implant devices and it is more common in older or immunosuppressed individuals (Cone et al., 2001; Chougle and Narayanaswamy, 2004; Kesteman et al., 2007; Charlier et al., 2012; Del Pozo et al., 2013; Chavada et al., 2014; Bush et al., 2015; Bader et al., 2016).

*Listeria monocytogenes* associated prosthetic joint infections (PJI) are on the rise probably in part due to the upsurge in prosthetic joint replacements in listeriosis high-risk groups (Charlier et al., 2012; Bush et al., 2015; Bader et al., 2016; Lenguerrand et al., 2018). Such infections are associated with substantial morbidity, impaired joint function, and at times limb amputation (Bush et al., 2015; Lenguerrand et al., 2018). Several antibiotic treatment regimens are reported with ampicillin or amoxicillin, either alone or in combination with gentamicin, being the most commonly used antibiotics (Charlier et al., 2012; Bush et al., 2015). Treatment duration is variable ranging from two weeks to as long as 18 months with some patients being placed on life-long oral antibiosis (Charlier et al., 2012; Bush et al., 2015). Moreover, treatment failures are also quite frequent among *Listeria* PJI cases receiving antibiotic therapy alone without surgical removal of the infected prosthesis (Charlier et al., 2012; Bush et al., 2015; Bader et al., 2016).

Osteoarticular listeriosis may become more common as the size of the population having specific risk factors related to this condition increases (Bader et al., 2016). Thus, further knowledge of *L. monocytogenes* involvement in this disease is necessary. We examined two *L. monocytogenes* strains that were isolated five years apart from a case of recurrent hip PJI in an 84-year-old patient who also received antibiotic treatments for the PJI and chronic obstructive pulmonary disease (COPD) during that period. Strains N843_10 isolated in 2010 and its recurrent strain N843_15 isolated in 2015 were phenotypic variants of the same *L. monocytogenes* strain. These two strains were characterized using phenome and genome-based approaches to investigate the physiological and molecular adaptive changes that might have evolved during long-term exposure to the human hip prosthetic joint environment resulting in these two phenotypic variants of the same *L. monocytogenes* clone.

## Materials and Methods

### Bacterial Strains, Genetic Manipulations, and Culture Conditions

Table 1 lists the strains and plasmids used in this study. Strains N843_10 and N843_15 were isolated by the National Center for Enteropathogenic Bacteria and Listeria (NENT) Switzerland. The *ΔrnjA* mutants were created through in-frame deletion of the *rnjA* (*lmo1027*) gene. A deleted copy for this gene retaining the reading frame, first six and last ten codons, as well as 500 bp of the upstream and downstream flanking sequences was synthesized based on the N843_10 genome sequence (GenScript Biotech, Netherlands). The Δ*rnjA* DNA fragment was cloned into the pKSV7 plasmid via the EcoR1 and Sa1I sites and used to replace the *rnjA* chromosomal copies in N843_10 and N2306 strains by homologous recombination as previously described (Smith and Youngman, 1992; Camilli et al., 1993; Schmid et al., 2009). N843_10 and N2306 *rnjA* locus deletion mutants were confirmed through PCR analysis and DNA sequencing. The Green fluorescent protein (GFP) and mOrange2 fluorescent protein (mO2FP) expressing strain variants were created through site specific PSA-integrase mediated single copy integration of the pPL3-eGFP (Shen and Higgins, 2005) and pIMK-mO2FP plasmids (unpublished and kindly provided by the Loessner lab, Laboratory for Food Microbiology, ETH Zurich) into the tRNA-Arg locus (Lauer et al., 2002; Monk et al., 2008). Bacteria were stored at −80°C in brain heart infusion medium (BHI, Oxoid, United Kingdom) supplemented with 20% glycerol. Strains were initially grown overnight on blood agar or BHI agar plates at 37°C to obtain single colonies, and then cultured twice in 10 ml BHI broth (37°C, 150 rpm) for 16 h generating stationary phase cultures that were routinely used as a starting point for experiments unless otherwise stated.

**TABLE 1:** Strains and plasmids used in this study

**Strain ID**	**Description**	**References/Source**
N843_10	2010 PJI isolate, serotype 1/2a, CC412	This study
N843_15	2015 PJI isolate, serotype 1/2a, CC412	This study
EGDe	Rabbit isolate and reference strain, serotype 1/2a, CC9	Glaser et al., 2001
N12-1273	Sporadic human listeriosis isolate, serotype 1/2a, CC412	Althaus et al., 2014
LL195	1983 Swiss listeriosis outbreak, serotype 4b, CC1	Bille, 1990
N2306	2013-2014 Swiss listeriosis outbreak, serotype 4b, CC4	Stephan et al., 2015
J5051	*Listeria innocua* used as anegative control	Guldimann et al., 2015
N11-1850	2011 milk isolate, serotype 4b, CC217, used as positive control in biofilm assays	Ebner et al., 2015
N843_10Δ*rnjA*	In-frame *rnjA* deletion	This study
N2306 Δ*rnjA*	In-frame *rnjA* deletion	This study
*E. coli* DH5α	Subcloning Efficiency^TM^ DH5α^TM^ competent *E. coli* competent cells	Invitrogen^TM^
**Florescent labeled *Listeria* strains**		
EGDe mO2FP	EGDe WT with pIMK-*mO2fp* integration into the tRNA^Arg^ locus	This study
N843_10 GFP	N843_10 with pPL3e-gfp integration into the tRNA^Arg^ locus	This study
N843_10 mO2FP	N843_10 with pIMK-*mO2fp* integration into the tRNA^Arg^ locus	This study
N843_15 mO2FP	N843_15 with pIMK-*mO2fp* integration into the tRNA^Arg^ locus	This study
N12-1273 mO2FP	N12-1273 WT with pIMK-*mO2fp* integration into the tRNA^Arg^ locus	This study
N2306 mO2FP	N2306 WT with pIMK-*mO2fp* integration into the tRNA^Arg^ locus	This study
*L. innocua* mO2FP	*L. innocua* with pIMK-*mO2fp* integration into the tRNA^Arg^ locus	Loessner Lab
N843_10Δ*rnjA* mO2FP	N843_10 Δ*rnjA* with pIMK-*mO2fp* integration into the tRNA^Arg^ locus	This study
N2306 Δ*rnjA* mO2FP	N2306 Δ*rnjA* with pIMK-*mO2fp* integration into the tRNA^Arg^ locus	This study
**Plasmids**		
pKSV7	Plasmid vector	Smith and Youngman, 1992
pKSV7-Δ*rnjA*	Plasmid carrying construct for *rnjA deletion*	This study
pPL3e-gfp	Integrative plasmid vector pPL3e—gfp for the constitutive expression of green fluorescence protein (GFP)	Shen and Higgins, 2005
pIMK-*mO2fp*	Integrative plasmid vector pIMK-*mO2fp* for the constitutive expression of mOrange2 fluorescence protein (mO2FP)	Loessner Lab

### Quantification of Cell Growth

Stationary cultures prepared from each strain as described above were diluted (1:100) in 10 ml BHI (10^7^ CFU/ml) and incubated at 37°C and 150 rpm. Growth was monitored by viable cell counting and OD_600_ measurement at defined time points. The experiments performed in duplicate were repeated on three separate occasions. The program DMFit (Baranyi and Roberts, 1994) was used to estimate growth parameters (lag phases and growth rates) from the OD_600_ data.

### Multilocus Sequence Typing (MLST) and Serotyping

MLST based on seven housekeeping gene fragments was performed as described on the Institut Pasteur website^1^ (last accessed 27 August 2019). The isolates were serotyped with *Listeria* O and H antisera (*Listeria* Antisera set, Denka Seiken co., Ltd, Japan), according to the manufacturer’s recommendations and PCR-serogroups were determined using a previously described multiplex PCR assay (Doumith et al., 2004).

### Microscopy

100 μl of overnight cultures grown in BHI broth, were fixed onto objective slides, Gram stained and processed for microscopic analysis. Slides were examined and photographed with a Leica DM4000B digital microscope through a 100x/1.3 oil-immersion objective. For electron microscopy, cells were harvested from overnight BHI cultures by centrifugation (6 000 *g* at 25°C for 5 min), fixed for 2 h at room temperature in 2.5% glutaraldehyde (Electron Microscopy Sciences) buffered in 0.1 M Sodium phosphate buffer (Sigma, Buchs, Switzerland), washed 3 times (0.1 M sodium phosphate buffer), fixed, and stained with 1% osmium tetroxide (Sigma, Buchs, Switzerland). Samples were dehydrated in ascending concentrations of ethanol followed by dehydration with propylene oxide (Sigma, Buchs, Switzerland) and infiltration in 30% and 50% Epon (Epoxy embedding medium, Sigma, Buchs, Switzerland). From each cell pellet, 0.9 mm toluidine blue stained semi thin sections were produced. Representative areas were trimmed and subsequently 90 nm, lead citrate (Merck, Germany) and uranyl acetate (Serva Electrophoresis, Baden-Würtenberg, Germany) contrasted ultrathin sections were produced and viewed under a transmission electron microscope (TEM Phillips CM10) at the Institute for Veterinary Pathology, Zurich, (IVPZ) at the University of Zurich.

#### Flow Cytometric Analysis

For this analysis, mOrange2 labeled strains (Table 1) were used. 750 μl of the overnight cultures of each strain were mixed with 250 μl of 4% paraformaldehyde and incubated at room temperature for an hour to fix the bacteria. Analysis of the strains was done at the Flow Cytometry Center (University of Zurich) using an Amnis ImageStream X Mk II imaging flow cytometer (Luminex, United States) fitted with a 488 nm excitation laser. Channel 3 that detects fluorescence between 560 to 595 nm was applied for the assay. Ten thousand events were counted for each sample and data was analyzed using the Image Data Exploration and Analysis software (IDEAS) version 6.2.

### Phenotypic Microarray Analysis

Strains N843_10 and N843_15 were analyzed for carbon source utilization (PM01 and PM02) as well as resistance to osmotic and pH stress (PM09 and PM10) on Biolog Phenotype Microarrays (PM) (Bochner, 2009^2^). The full list of the tested compounds can be obtained from https://www.biolog.com/wp-content/uploads/2020/04/00A-042-Rev-C-Phenotype-MicroArrays-1-10-Plate-Maps.pdf (accessed July 20, 2020). The experiments were performed in duplicate at 37°C following standard Biolog Inc., protocols with a few modifications as previously described (Muchaamba et al., 2019).

### Motility Assay

Five microliter of overnight BHI culture (1 × 10^9^ CFU/ml) of the different strains was spot inoculated on the surface of soft BHI agar (0.25% agar) plates containing 0.05% Triphenyltetrazolium Chloride (TTC) and incubated at 25°C for 48 h. Motility was determined by measuring the diameter of the red zone created by the spreading colony.

### Antibiotic Sensitivity

Before each experiment, bacteria were plated by streaking on blood agar plates and grown overnight at 37°C. Tests for antibiotic susceptibility using commercial E test strips against a panel of 8 antimicrobials: amoxicillin and clavulanic acid (Amoxy Clav), cephalothin, tetracycline, ciprofloxacin, penicillin G, sulfamethoxazole, azithromycin and gentamicin, were done in accordance with the manufacturers recommendations (Biomerieux, Lyon, France; Liofilchem, Italy). Briefly, bacteria solutions of 0.5 McFarland standard density were plated to form a lawn on the surface of a Muller Hinton plus blood agar plate. An appropriate antibiotic *E* test strip was then placed on respective plates and results were assessed after 48 h of incubation at 37°C. The minimum inhibitory concentration (MIC) was defined as the highest concentration at which the colonies touched the *E* test strip.

### Biofilm Assays

Biofilm experiments were performed in Tryptone soy broth (TSB) medium at 37°C. Overnight aliquots of TSB secondary cultures prepared as described above were added (1:40) to fresh TSB medium then 100 μl of this mixture was added to 96-well microtiter plates. Biofilms were grown for 96 h at 37°C. Unbound cells were removed by washing three times with 150 μl sterile deionized water then inversion and tapping of the 96 well plate on absorbent paper. Microplates were dried at 37°C for 30 mins, and adherent cells stained with 150 μl aqueous crystal violet for 20 min. Excess stain was removed by 5 washings with deionized water. The bound stained cells were quantified by addition of 96% ethanol to dissolve the crystal violet and the dissolved stain was measured at an optical density of 595 nm using a Synergy HT OD reader (Biotek Instruments, GmbH, Switzerland). Each biomass was standardized relative to *L. monocytogenes* N11-1850.

### Cell Invasion, Hemolysis and Phosphatidylinositol-Specific Phospholipase C (PI-PLC) Assays

Cell invasion assays using the human enterocyte-like Caco-2 (ATCC^®^ HTB-37^TM^) cell line and hemolysis using human red blood cells were performed as previously described (Muchaamba et al., 2019). To compare PI-PLC activities the strains were grown on OCLA (Oxoid Chromogenic Listeria Agar) plates. Overnight BHI cultures of each strain were serially diluted in phosphate buffered saline (PBS; 10^5^, 10^4^ and 10^3^ CFU/ml) and spotted (10 μl) on OCLA plates that were incubated at 37°C and visually examined after 48 h for the zone of opacity. *L. monocytogenes* LL195 and *L. innocua* JF5051 strains were included as positive and negative controls, respectively. Experiments were conducted in triplicate on three separate occasions.

### Zebrafish Microinjection and Localized Infection Assays

Zebrafish husbandry and assays were performed using the *Danio rerio wik* zebrafish line strains as previously described (Eshwar et al., 2017). Bacteria for microinjection experiments were harvested from secondary stationary phase stage BHI cultures prepared as described above by centrifugation, washed once and diluted to 5 x 10^8^ CFU/ml in DPBS. Two-day post fertilization embryos were injected with approximately 500 CFU in 1–2 nl volume of a bacterial suspension in DPBS into the blood circulation via the caudal vein. Injected CFU numbers were controlled through viable cell counting performed on the microinjection DPBS droplet and five individual embryos immediately disintegrated after microinjection. Post-infection embryos were placed into 24-well plates (one embryo per well) in 1 ml E3 medium per well, incubated at 28°C and observed under a stereomicroscope twice a day up to 72 h post infection (hpi) for signs of disease including developmental delay (especially swim bladder and decreased locomotory rate), necrosis at the inoculation site, subsequent necrosis in other parts of the body, and eventual death. The number of dead larvae was determined visually based on the absence of a heartbeat. The *Danio rerio wik* zebrafish Fli1: GFP and *Danio rerio wik mpeg1*: GFP zebrafish line strains with GFP-labeled endothelial cells and macrophages, respectively, were used for the localized infection experiments. Fluorescent-labeled bacteria were prepared and harvested as described above. Two-day post fertilization embryos were injected with approximately 1200 CFU in 1–2 nl volume of a bacterial suspension in DPBS into the otic vesicle to simulate a localized infection. The number of CFU injected and post-infection embryo husbandry was controlled as described above. The embryos were monitored for signs of disease and survival under a stereomicroscope at 8, 24, 48, and 72 hpi. The number of dead larvae was determined visually based on the absence of a heartbeat. At each time point, 10 embryos were fixed and prepared for confocal microscopy. 3D-image stacks of whole mount samples were prepared using a confocal laser-scanning microscope (CLSM, Leica TCS SP8, Leica Microsystems, Heerbrugg, Switzerland). GFP and mOrange were sequentially excited with the 488 nm and 561 nm laser lines, respectively, with emission signals collected within the respective range of wave lengths. 3D image stacks were collected sequentially (to prevent green–red channel crosstalk) according to Nyquist criteria and deconvolved using HuygensPro via the Huygens Remote Manager v2.1.2 (SVI, Netherlands). Images were further analyzed with Imaris 7.6.1 (Bitplane, Zurich, Switzerland). Furthermore, two-day post fertilization zebrafish Fli1: GFP embryos were injected with approximately 2400 CFU in 2 nl volume of a 1:1 mixture of GFP labeled N843_10 and mOrange labeled N845_15 bacterial suspension in DPBS into the otic vesicle to simulate a coinfection. The number of CFU injected and post-infection embryo husbandry was controlled as described above. Plating on selective media to enumerate the individual coinfected strains was done at 0, 8, 24, and 48 hpi.

### Genome Analysis

Genomes of *L. monocytogenes* N843_10 (CP046361), N843_15 (CP046362), N12-1273 (QYFZ00000000), EGDe (NC003210), LL195 (HF558398), Lm3136 (CP013723), Lm3163 (CP013722), N1546 (CP013724), N2306 (CP011004), N16-0044 (CP035187), EGD (HG421741), 10403S (NC_017544), Clip 804259 (AE017262), and F2365 (NC002973) were used in this study. Genomic DNA isolated from the strains N843_10 and N843_15 using the GenElute Bacterial Genomic DNA Kit (Sigma, Buchs, Switzerland) was sequenced using the Pacific Biosciences single-molecule real-time sequencing technology (SMRT) and assembled *de novo* using the SMRT Analysis 2.3.0 software (ChunLab, Seoul National University). Rapid Annotation Subsystem Technology (RAST) and Seed Viewer^3^ were used for genome annotation and comparisons. Gepard was used for the dot-plot analysis of the genomes (Krumsiek et al., 2007). MAUVE was used to align the genomes and to derive the coordinates for the positions of the single nucleotide polymorphisms (SNPs), insertions and deletions (InDels) (Darling et al., 2010). Genes of interest were extracted and compared between the genomes using CLC genomics Workbench (Qiagen, Prismet, Denmark) and the BLASTn and BLASTp programs (blast.ncbi.nlm.nih.gov/Blast.cgi). Core genome MLST (cgMLST) analysis was performed using the software package Seqsphere+ 6.0 (Ridom GmbH, Münster, Germany). Complete genomes sequences of N843_10 and N843_15 were compared with a local database containing 567 *L. monocytogenes* genomes (ILS, unpublished data). The MLST sequence type was determined according to the scheme of the Institut Pasteur^4^; cgMLST according to scheme at cgMLST.orgm^5^ (Ruppitsch et al., 2015). Minimal spanning trees were constructed using Seqsphere+ with the option “missing values pairwise ignored.” Relatedness of the two strains (N843_10 and N843_15) was further assessed by SNP comparisons including a selection of other unrelated reference strains. SNPs were identified using parsnp within the harvest suite (Treangen et al., 2014) using standard settings and nucleotide fasta files as input. Each strain was used as a reference strain and compared to the other strains. The output files were converted to variant calling files using harvesttools and a SNP matrix was constructed by taking the sum of the variants compared to the reference strain. The SNP matrix was visualized in a heatmap using clustvis (Metsalu and Vilo, 2015). DuctApe software was used to compare genomes in correlation with PM data, using it to detect genes encoding enzymes that could be involved in the metabolic pathways responsible for the phenotypes observed in carbon source utilization on PM01 and PM02 (Galardini et al., 2014). Genes described in the Kyoto Encyclopedia of Genes and Genomes (KEGG) database were considered in this approach. Mutations in selected genes uncovered in WGS were further confirmed through Sanger sequencing at Microsynth AG (Balgach, Switzerland; Supplementary Table S1).

### RNA Isolation and Reverse Transcription Quantitative PCR (RT-qPCR)

Secondary bacterial cultures prepared as described above were diluted (1:100) in 50 ml and grown to the late exponential phase (OD_600_ of 1.4; 10^9^ CFU/ml). Bacteria (1 ml) were harvested in RNA protect Bacteria reagent (Qiagen GmbH, Hilden, Germany) and total RNA was isolated using the RNeasy Plus Mini Kit (Qiagen GmbH, Hilden, Germany) as previously described (Eshwar et al., 2017). RNA yield and quality were assessed using the Quanti Fluor RNA System (Promega, Madison, United States) and the BioAnalyzer (Agilent Technologies, United States). RNA (400 ng and RIN ≥ 7.8) was converted to cDNA using the Quantitect Reverse Transcription Kit (Qiagen GmbH, Hilden, Germany). Samples (2.5 ng cDNAs) were amplified using primers listed in Supplementary Table S1 and the SYBR green I kit (Roche Molecular Diagnostics GmbH, Mannheim, Germany) in the Light Cycler LC480 instrument (Roche Molecular Diagnostics, Rotkreuz, Switzerland). Relative mRNA quantification was performed using the Light Cycler 480 Relative Quantification Software (Roche Molecular Diagnostics). mRNA amounts were normalized using 16S rRNA and expressed relative to a calibrator mRNA sample derived from a stationary phase *L. monocytogenes* EGDe culture.

### Statistical Analyses

All experiments presented were performed independently in duplicate at least three times unless stated otherwise. GraphPad Prism (Version 8.3.0 (328), GraphPad Software, San Diego, CA, United States) was used for the statistical analysis of data. One-way ANOVA with post-hoc Tukey HSD tests was used to assess the statistical significance of differences between the strains. *P*-values < 0.05 were considered to be statistically significant. PM data was analyzed using the programs ductApe and opm (version 1.3.64) (Galardini et al., 2014; Göker et al., 2016). Area under the curve was the reference parameter for opm analysis, whereas for DuctApe the parameter, activity index (AV) was used as previously described (Galardini et al., 2014; Göker et al., 2016). For each compound tested, the final result was expressed as the mean of two replicates. The bacterium was not able to respire under conditions were AV value was equal to zero, whilst it was able to respire under conditions were the AV values were higher than zero.

## Results

### N834_15 and N843_10 Are Phenotypic Variants of the Same *L. monocytogenes* Strain Isolated Five Years Apart From a Recurrent Human Hip PJI

**FIGURE 1:**
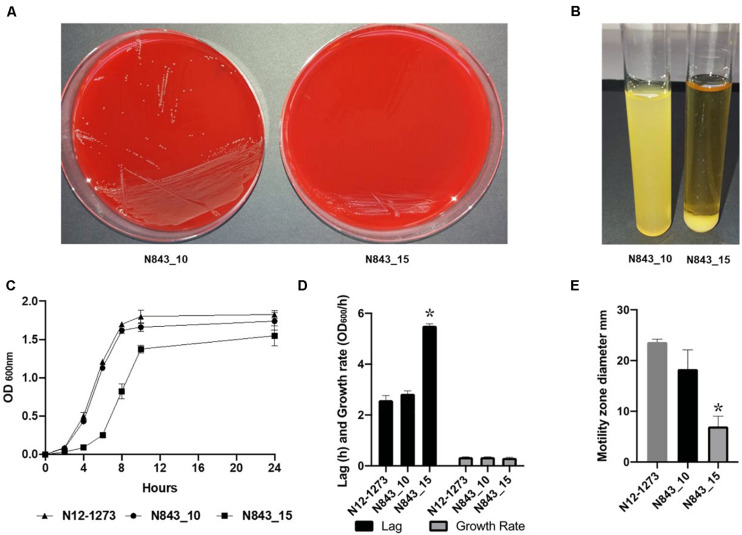
N843_15 exhibits slower growth, atypical aggregation behavior and reduced motility compared to N843_10. **(A)** Growth of N843_10 and N843_15 on blood agar incubated 24 h at 37°C. **(B)** N843_15 aggregated and sedimented upon growth in BHI broth. **(C and D)** Comparison of N843_15, N843_10 and N12-1273 growth kinetics in BHI broth at 37°C. **(E)** N843_10, N843_15 and N12-1273 swarming motility zones determined on semi-solid BHI agar at 25°C. Results presented are the means and standard deviations from three independent experiments. ^∗^ Indicates statistically significant difference between the strains (*P* < 0.05).

**FIGURE 2:**
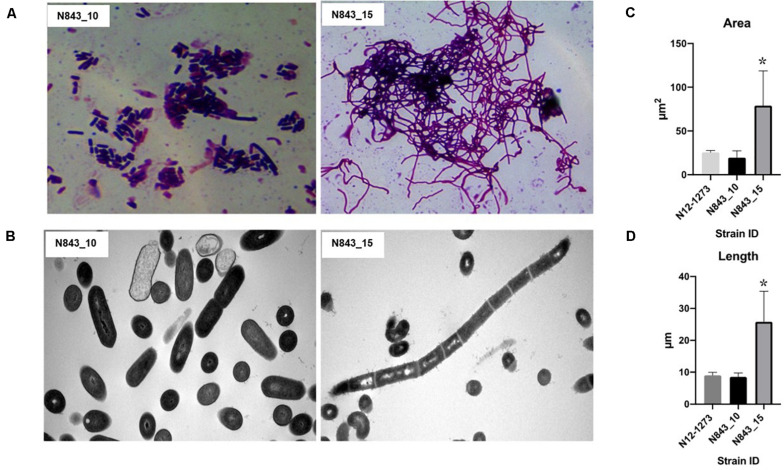
N843_10 and N843_15 cells display morphological differences. **(A)** N843_15 is filamented whereas N843_10 occurs in single rods and short chains under the light microscope. **(B)** Electron microscopy (EM) images showing that N843_15 filamentation phenotype is due to chains of unseparated cells whilst N843_10 showed single rods and short chains typical of *L. monocytogenes* morphology. **(C and D)** Flow cytometry analysis revealed significantly increased area and cell chain length in N843_15 compared to N843_10 and the clonal control strain N12-1273 (^∗^*P* < 0.05).

**FIGURE 3:**
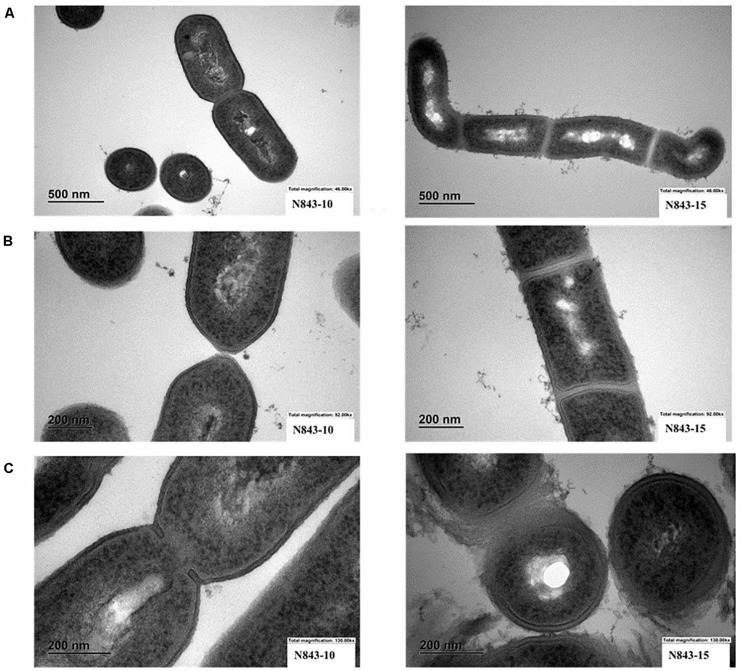
Electron microscopy (EM) reveals variations between N843_10 and N843_15 cell morphology. **(A)**. N843_10 displays typical *L. monocytogenes* morphology of rod-shaped cells whereas the filamented N843_15 cells varied in shape **(B)** N843_10 cells displayed normal cell wall morphology and separation during cell division whilst N843_15 cells displayed altered cell wall morphology and lacked clear cell division septum demarcation. **(C)** N843_10 has a normal cell wall and is undergoing normal septum formation whereas N843_15 has altered cell walls with loosely attached fragments of peptidoglycan protruding from its surface giving it a solar flare appearance.

**FIGURE 4:**
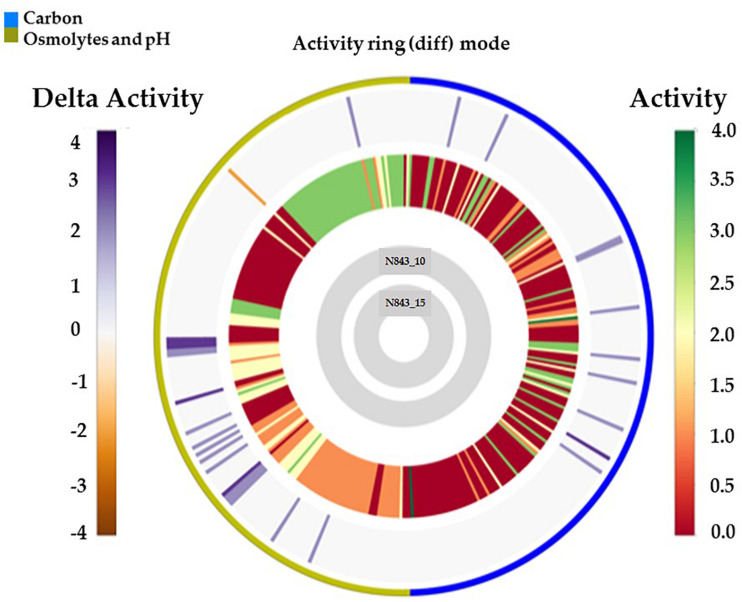
Overall growth or metabolic activity ring comparison of N843_15 and N843_10 with respect to C-source utilization (PM01 and PM02) and stress (osmolytes and pH; PM09 and PM10) resistance based on phenotypic microarray analysis. The gray inner circles indicate the strains’ order whilst the external circle indicates the PM categories. The metabolic activity referred to as activity index (AV) calculated for N843_15 under each assay condition per well is reported as color stripes going from red (AV = 0, no metabolism) to green (AV = 4, highest metabolic activity). Delta activity: the difference in the metabolic activity (AV) of N843_10 and N843_15 is reported when equal to or higher than 2 AV; gray is no difference; purple indicates a higher metabolic activity of N843_10 whilst orange color indicates that N843_10 has a lower metabolic activity than N843_15 under the assay conditions in that well.

We isolated two *L. monocytogenes* strains from recurrent human hip PJI episodes recorded five years apart in 2010 (N843_10) and 2015 (N843_15), respectively. Typing of these two isolates based on PCR and seven loci multi-locus sequence typing (MLST) assigned them both to PCR serogroup IIa, MLST sequence type (ST) ST412 and clonal complex (CC) CC412. As such the original (N843_10) and relapse (N843_15) PJI isolates were clonal descendants and derivatives of the same *L. monocytogenes* strain responsible for the recurrent PJI episodes recorded five years apart. On phenotypic analysis these two isolates were however, phenotypically distinct since compared to the initial PJI isolate N843_10, the recurrent infection isolate N843_15 grew slower on both solid and liquid media as well as aggregated and sedimented in BHI broth (Figures 1A–D). In a swarming motility assays the isolate N843_15 was also significantly less motile than N843_10 (Figure 1E). Both light and electron microscopy examination of liquid and pelleted stationary phase cultures, respectively, revealed that while all N843_15 cells observed occurred in long filamented chains those of N843_10 retained typical *L. monocytogenes* cell morphology comprising single rods to short chains (Figures 2A,B). Consistent with filamentation in this strain, flow cytometric analysis also revealed increased area and chain length for N843_15 cells compared to N843_10 cells (Figures 2C,D). Electron microscopic examination further revealed improper cell septum formation and incomplete separation suggestive of cell division defects in N843_15 (Figures 3A–C). Cells of this isolate were highly irregular arranged in long chains of varying lengths without a well-defined cell structure, whereas the peptidoglycan layer was disordered with loosely attached fragments projecting from the surface giving it a solar flare appearance in contrast to a well-organized peptidoglycan observed for N843_10. Further physiological comparison of the two isolates using Biolog phenotypic microarray revealed reduced carbon source utilization range as well as increased osmotic and pH stress sensitivity in N843_15, the relapse PJI isolate compared to the initial isolate N843_10 (Table 2 and Figure 4). N843_15 had lost the ability to utilize 13 C-sources including relevant intracellular C-sources such as glycerol, maltose, and cellobiose (Table 2, Supplementary Table S2 and Supplementary Figure S1). Although overall more sensitive to various stress conditions, N843_15 was more resistant to alkaline stress (pH 9.5) than N843_10 in presence of β-Phenylethylamine (Table 2). Overall these analyses thus showed that N843_10 and N843_15 were phenotypic variants of the same *L. monocytogenes* strain, which suggested that N843_10, the initial PJI strain had evolved within the human hip PJI environment giving rise at some point to strain N843_15 isolated five years later during the relapse infection.

**TABLE 2:** Comparison of carbon source utilization ability, pH and osmotic stress tolerance.

**Intracellular relevant carbon sources**	**N843_15**	**N843_10**	**Food relevant carbon sources**	**N843_15**	**N843_10**
Glycerol	−	**+**	Pectin	−	**+**
D-Maltose	−	**+**	D-Arabinose	−	**+**
D-Cellobiose	−	**+**	Palatinose	−	**+**
2-Deoxy-D-Ribose	−	**+**	D-Tagatose	−	**+**
Thymidine	−	**+**	**Osmotic and pH stress**		
Adenosine	−	**+**	Sodium chloride	−	**+**
Inosine	−	**+**	Sodium lactate	−	**+**
**Others**			Sodium benzoate at pH 5.2	−	**+**
3-*O*-Methyl-D-Glucose	−	**+**	Sodium Nitrite	−	**+**
5-Keto-D-Gluconic Acid	−	**+**	β-Phenylethylamine at pH 9.5	**+**	−

*Symbols: −, negative reaction; **+**, positive reaction. Opm generated results showing phenotypic variability on different carbon sources as well as under different pH and osmotic stress conditions among the two isolates. N843_10 utilized the most carbon sources and showed the highest metabolic activities under pH and osmotic stress conditions than N843_15.*

### N843_15 the Recurrent PJI Isolate Displays Increased Antibiotic Sensitivity and Reduced Biofilm Production Than the Original Infection Parent Isolate N843_10

**FIGURE 5:**
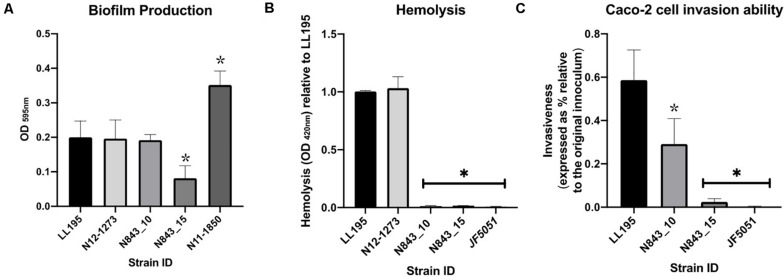
Biofilm production, hemolysis and Caco-2 cell invasion. **(A)** N843_10 produced more biofilm than N843_15. **(B)** *L. monocytogenes* N843_10 and N843_15 were poorly hemolytic compared to LL195 (CC1) and N12-1273 (CC412) strains **(C)** N843_15 showed lower cell invasion capacity than N843_10 but both strains were significantly less invasive than the reference strain LL195. Presented data shows the mean (bars) and standard deviation (error bars) of three independent biological experiments. ^∗^Indicates statistically significant differences where *P* < 0.05 based on one-way ANOVA and Tukey post-hoc test pairwise comparison of all the strains.

Assuming changes in either antibiotic susceptibility or biofilm production ability could have facilitated the long-term persistence and survival of the two isolates in the PJI contributing to emergence of the evolved variant N843_15, the antibiotic susceptibilities and biofilm production of the two PJI isolates were compared. Both strains were clinically sensitive to different therapeutic antibiotics associated with antibiotic therapy in the patient as well as gentamicin and sulfamethoxazole. The recurrent infection isolate N843_15 although more sensitive than the parent strain on most of the tested antibiotics was, however, more resistant to sulfamethoxazole (Table 3). N843_15 produced significantly less biofilm than the parent isolate N843_10 when assessed in TSB at 37°C (Figure 5A). The overall biofilm production of the parent isolate N843_10 was similar to other *L. monocytogenes* strains tested such as LL195 (CC1) and N12-1273 (CC412) but significantly less in comparison to a high biofilm producer reference strain N11-1850 (CC217) (Figure 5A). Our findings thus indicated that increased antibiotic resistance or enhanced biofilm production were unlikely to have played a role in the emergence of the evolved recurrent infection N843_15 isolate from the initial PJI isolate N843_10.

**TABLE 3:** MICs of different antibiotics determined for the *L. monocytogenes* N843_10 and N843_15 strains using *E*-tests.

**Antibiotic^∗^**	**MIC of N843_10 (μg/ml)**	**MIC of N843_15 (μg/ml)**
Amoxicillin and Clavulanate^1^	0.420.07	0.100.02
Cephalothin	4.71.2	10
Azithromycin	0.590.36	0.290.1
Tetracycline	1.330.2	0.320.1
Ciprofloxacin	0.50	0.250
Gentamicin**^2^**	0.060.01	0.050
Penicillin G	0.50	0.090
Sulfamethoxazol	120	160

*Listed are antibiotics that the isolates could have been directly or indirectly exposed to during their residency in the joint. The antibiotics were given at different intervals in different protocols and via different routes in accordance to the physicians’ recommendations. **^1^**Treatment given on two different occasions once for 10 days for treatment of prosthetic joint listeriosis and on another instance for treatment of Chronic Obstructive Pulmonary Disease (COPD). **^2^**Gentamicin was included because it is a commonly used drug in listeriosis treatment.

### PJI Isolates N843_10 and N843_15 Are Less Virulent Compared to Other *L. monocytogenes* Strains

**FIGURE 6:**
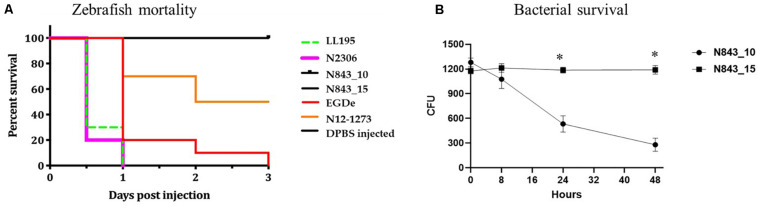
Strains vary in virulence and persistence within zebrafish: **(A)** In zebrafish embryos infected using the blood stream injection route, both N843_15 and N843_10 were unable to induce any clinical signs or mortality 72 h post infection. The reference strains used N2306 (CC4) and LL195 (CC1) had induced 100%, whilst EGDe (CC9) and N12-1273 (CC412) had induced 100% and 50% mortality of the zebrafish embryos within 24 and 72 h of infection, respectively. **(B)** GFP zebrafish embryo line coinfected with N843_15 mO2FP and N843_10 GFP. The parent strain N843_10 decreased in CFU numbers over time consistent with it being cleared while N843_15 levels remained unchanged over 48 h of infection. Results show the mean and standard deviation from 3 independent experiments. ^∗^Indicates significant differences in the level of the two strains at 24 and 48 hpi (*P* < 0.05 based on repeated measures two-way ANOVA).

**FIGURE 7:**
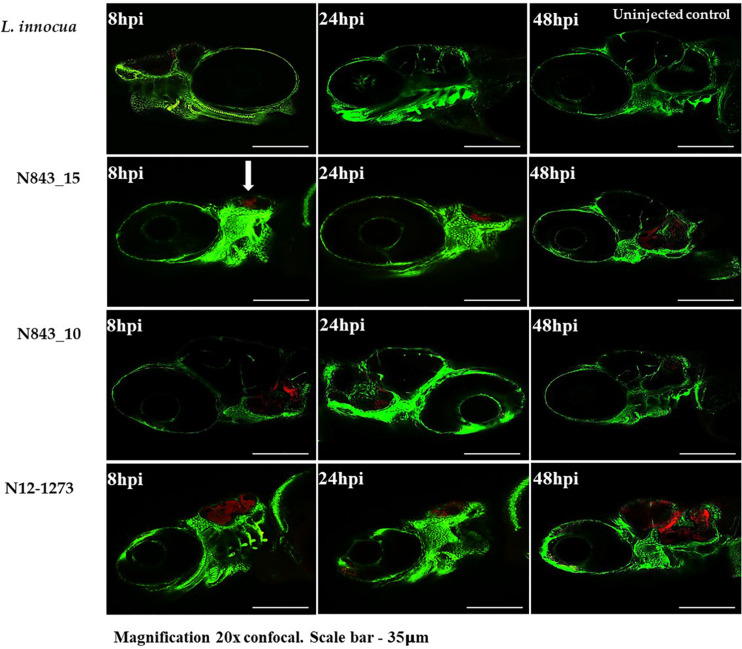
Localized infection simulation using *Danio rerio wik* zebrafish Fli1: GFP. N843_15 was not cleared by the immune system neither did it cause diseases over 48 h of infection whereas N843_10 infection was cleared. N12-1273 used as a clonal positive control caused disease and was not cleared 48 hpi whilst the *L innocua* negative control used was already cleared at 24 hpi. Arrow denotes the infection site (otic vesicle) and the bacteria are in red (Magnification 20× confocal. Scale bar – 35 μm).

A virulence comparison of the two PJI isolates showed that they had similar hemolytic activity levels on human red blood cells but the relapse infection isolate N843_15 was impaired in human Caco 2 cell invasion compared to the parent PJI isolate N843_10 (Figures 5B,C). On the other hand, both isolates showed significantly lower hemolytic and cell invasion when compared to other *L. monocytogenes* strains including another MLST clonal complex CC412 sporadic human listeriosis isolate N12-1273, as well as a CC1 previous listeriosis outbreak strain LL195 (Figures 5B,C). Comparing virulence using a zebrafish embryo-based infection model showed that both PJI isolates were avirulent whereas other strains used as positive controls such as *L. monocytogenes* EGDe, N12-1273, N2306 and LL195 were virulent causing mortality (Figure 6A). In a simulated localized zebrafish embryo infection model both PJI isolates similar to *L. innocua* the negative control were unable to induce disease signs over 48 h. Interestingly, while the injected N843_10 cells were cleared by the immune system those of the relapse isolate N843_15 remained uncleared nor did they decrease in quantity at the infection site (Figures 6B, 7). On the other hand, the CC412 clonal positive control strain N12-1273 was able to spread from the injection site and induce disease signs. Furthermore, the cells of this strain were not cleared by the immune system 48 hours post infection (hpi) but those of the *L. innocua* negative control had been cleared already at 24 hpi (Figure 7).

### Genome-Wide Comparison Reveals Genetic Changes in the Evolved PJI Isolate *L. monocytogenes* N843_15

**FIGURE 8:**
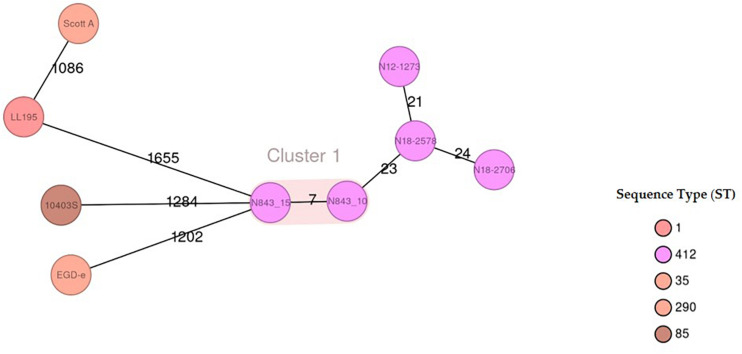
Minimum-spanning tree illustrating the phylogenetic relationship based on cgMLST allelic profiles of the 2 PJI isolates (N843_10 and N843_15) and seven selected reference strains including three CC412 isolates (N12-1273, N18-2578 and N18-2708). Each circle represents an allelic profile based on sequence analysis of 1,701 cgMLST target genes. The numbers on the connecting lines illustrate the numbers of target genes with differing alleles. The different groups of strains are distinguished by the colors of the circles. Clonal strains (cutoff 10 allele difference) are shaded in light brown.

To assess if the different phenotypes between N843_10 and N843_15 were associated with genetic changes during *L. monocytogenes* N843 evolution within the human prosthetic joint environment we used whole genome sequences (WGS) analysis and compared the two N843 isolates. An *in silico* classical (seven loci) and core genome based MLST analysis both confirmed the two isolates to belong to MLST sequence type ST412 and clonal complex CC412. In addition, the N843_10 and N843_15 genomes clustered indistinguishably in a WGS-based phylogenetic tree and aligned colinearly without major chromosomal differences in a DNA sequence dot blot (Supplementary Figures S2A,B). Overall the two isolates showed only seven cgMLST allelic differences that are below the 10-allele difference recommended as the strain clonality cut off for *L. monocytogenes*. In contrast there were 21-24 and 1086-1655 cgMLST allelic profile differences when the two PJI isolates were compared to other epidemiologically unrelated ST412 strains (N12-1273, N18-2578 and N18-2708) and selected *L. monocytogenes* reference strains (EGDe, 10403S, ScottA and LL195) from other MLST STs, respectively (Figure 8, Supplementary Figure S3). Overall there were 26 genome wide single nucleotide polymorphisms (SNPs) detected in N843_15 compared to N843_10, which included 9 SNPs that cause non-synonymous amino acid changes in proteins with known functions (Table 4, Supplementary Table S3). While the other SNPs were located in intergenic regions and hypothetical proteins their potential functional consequences remain unknown. In addition, there were 44 insertion and deletion events (InDels) detected in the N843_15 genome including 21 InDels that are predicted to cause premature stop codons (PMSC) and truncation of proteins such as RNase J1 and HtrA (Table 5, Supplementary Table S3). A single base deletion was detected in the *prfA* gene of both N843_10 and N843_15 compared to *L. monocytogenes* EGDe *prfA*, which is predicted to cause a PMSC and an eleven amino acid PrfA carboxyl-terminal truncation (Supplementary Figure S4).

**TABLE 4:** Single nucleotide polymorphism detected between N843_15 and N843_10 genomes and their predicted consequences^1^.

**Gene name^2^**	**aa change^3^**	**Side chain class, polarity and charge change^3^**	**Description of affected protein**
*lmo0243*	A753D	Aliphatic, nonpolar, neutral to acid, acidic polar negative	DNA-dependent RNA polymerase (EC 2.7.7.6)
*lmo0972 (dltC)*	V7I	Similar	D-alanine-poly (phosphoribitol) ligase subunit 2 (EC 6.1.1.13)
*comEC*	V25I	Similar	Late competence protein ComEC
*lmo1586 (ppnk)*	L23S	Aliphatic non-polar neutral to hydroxyl containing polar neutral	NAD kinase (EC2.7.1.23)
*lmo2121*	I670M	Sulfur containing nonpolar neutral to aliphatic nonpolar neutral	Maltose phosphorylase
*lmo2591*	V15C	Aliphatic nonpolar neutral to sulfur containing nonpolar neutral	N-acetylmuramoyl-L-alanine amidase (Flg J hydrolase)
*lmo2596*	P76Q	Cyclic non-polar neutral to amide polar, neutral	30S ribosomal protein
*lmo2635*	R113S	Basic, basic polar positive to hydroxyl containing polar neutral	1,4-dihydroxy-2-naphthaloate octaprenyltransferase
*lmo2829*	C194R	Sulfur containing, nonpolar neutral to basic, basic polar, positive	Putative nitroreductase HBN1

*^1^Only those SNPs in coding regions of genes with known functions are listed. ^2^Based on *L. monocytogenes* EGDe genome annotation; ^3^Predicted amino acid change.*

**TABLE 5:** InDels detected in the N843_15 genome compared to N843_10^1^.

**Gene name^2^**	**Insertion or deletion**	**Length in N843_10^3^**	**Length in N843_15^3^**	**Description of changes in N843_15^4^**
*lmo0292*	A deletion	501	100	PMSC creating a truncated HtrA protein
*23S rRNA*	G deletion			Altered 23S rRNA sequence
*lmo0533*	ATG deletion	89	0	UPF0237 protein- methionine (start codon) lost from coding gene hence no translation
*lmo1027*	A deletion	556	443	aa change and PMSC leading to truncated Ribonuclease J protein
*clpQ*	A insertion	179	125	PMSC creating a truncated ATP dependent protease HsIV (E.C.3.4.25-)
*potA potB*	CATGAGT deletion	118 202	306	aa change and loss of stop codon – potA and potB of a putative ABC transporter fused
*lmo1799*	584 bp deletion	956	762	Truncated putative peptidoglycan bound protein (LPXTG motif)
*hly-III*	T-deletion	211	207	PMSC creating truncated hemolysin III protein
*16S rRNA*	C deletion			16S rRNA
*lmo2590*	AAA deletion	342	341	Lysine at position 131 deleted in Mrp/Nbp35 family ATP binding protein
*rplO*	A deletion	146	81	PMSC creating a truncated 50S ribosomal protein L15 subunit
*bvrA*	T deletion	689	46	L47PMSC creating a truncated predicted regulator of galactitol operon (BglG), (E.C.2.7.1.69) and aa changes N45T and W46G
*lmo0842*	9 bp insertion	2063	2066	3 aa insertion into putative peptidoglycan bound protein (LPXTG motiff)
*pyrG*	TTG insertion	553	533	PMSC creating truncated CTP synthase (E.C.6.3.4.2)
*rpsE*	A-deletion	167	145	PMSC creating truncated SSU ribosomal protein S5p (S2e)
*lmo2812*	A-insertion	272	258	K259PMSC creating truncated D-alanine carboxypeptidase (PBDB2) and aa changes R257T and F258V.
*arcD*	G deletion	270	245	PMSC creating truncated Arginine/Ornithine antiport protein

***^1^**Only those InDels affecting proteins with known functions are listed. ^2^Based on *L. monocytogenes* EGDe genome annotation; **^3^**length as amino acid number. ^4^Premature stop codon (PMSC).*

### Assessing the Impact of the PrfA Truncation Mutation Predicted in N843_10 and N843_15

**FIGURE 9:**
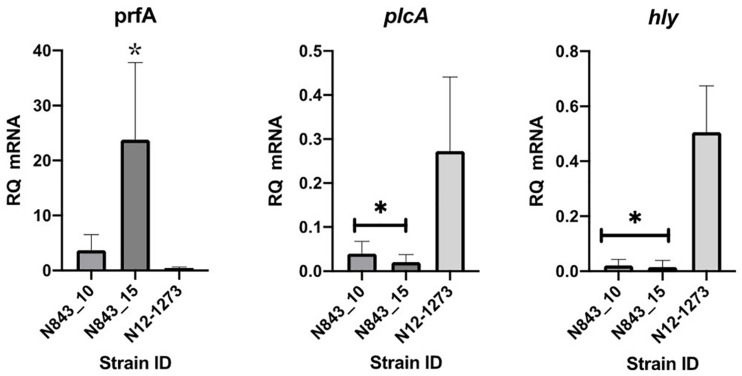
Impact of the PrfA truncation mutation predicted in N843_10 and N843_15 on virulence gene mRNA levels. Quantification of *prfA, plcA*, and *hly mRNAs* using qRT-PCR in the study strains that were cultured in BH broth at 37°C to the late exponential phase. Relative quantities (RQ) of *prfA, plcA*, and *hly* mRNA levels were normalized to 16S rRNA and are expressed relative to those of a *L. monocytogenes* EGDe based mRNA calibrator sample. Presented data shows the mean (bars) and standard deviation (error bars) of three independent biological experiments. ^∗^Indicates statistically significant differences where *P* < 0.05 based on one-way ANOVA and Tukey post-hoc test pairwise comparison of all the strains.

The phenotypic impact of the predicted PrfA truncation mutation in both PJI isolates was assessed through qRT-PCR analysis of *prfA* target genes showing that although containing more *prfA* mRNA, both N843_10 and N843_15 contained significantly low *hly* and *plcA* mRNA levels when compared to a clonal but epidemiologically unrelated positive control strain N12-1273 with an intact full length PrfA (Figure 9). The relapse PJI isolate N843_15 interestingly also contained higher *prfA* but lower *hly* and *plcA* mRNA compared to the parent strain isolate N843_10. Reduced *plcA* expression in N843_15 was also corroborated through qualitative PI-PLC activity analysis, which showed reduced activity in N843_15 than N843_10 (Supplementary Figure S5). PI-PLC activity of both PJI strains was, however, significantly lower than *L. monocytogenes* N12-1273 (CC412) and N2306 (CC4) (Supplementary Figures S5A,B). Overall these results were consistent with impaired PrfA activity in N843_10 and N843_15 strains confirming the WGS analysis predicted PrfA truncation mutation.

### Phenotypic Characterization of RNase J1 Mutants in *L. monocytogenes* N843_10 and N2306 Strains

**FIGURE 10:**
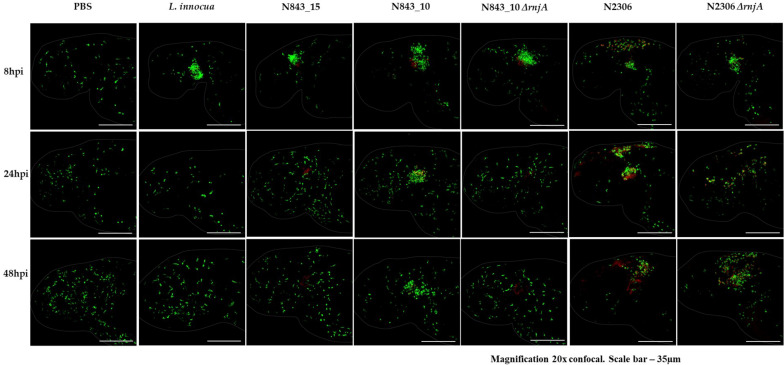
In the zebrafish *mpeg1*: GFP line infection with N843_15 and N843_10 *ΔrnjA*, macrophages were initially attracted to the injection site (otic vesicle) but returned to the normal distribution similar to PBS injected fish within 24 h without clearing the bacterial from the site of infection. Macrophages are green and the bacteria are in red (Magnification 20x confocal. Scale bar – 35 μm).

In order to assess phenotypic consequences of RNase J1 function loss due to a truncation mutation predicted in the relapse strain N843_15, *rnjA* gene deletion mutants in *L. monocytogenes* N843_10 (CC412) and N2306 (CC4) were created. These strains represented genetic backgrounds of the parent isolate (N843_10) and a different genetic lineage and MLST clonal complex (N2306), respectively. Recapitulating some of the phenotypic defects displayed in N843_15, both N843_10 and N2306 Δ*rnjA* mutants had reduced swarming motility, low *plcA* and *hly* mRNAs amounts and reduced PI-PLC activity, but showed increased cellular filamentation, antibiotic sensitivity and sulfamethoxazole resistance compared to their parental WT strains (Supplementary Figures S6–S8, Supplementary Table S4). Although the extent of filamentation in these Δ*rnjA* mutants was less pronounced than that of N843_15. In virulence evaluation using the simulated localized zebrafish embryo infection model, similar to N843_15, cells of N843_10 Δ*rnjA* were also not cleared by the immune system and did not cause detectable disease signs over 48 h of infection (Figure 10). As with the localized N843_15 infection macrophages were also initially attracted to the site of N843_10 Δ*rnjA* injection but returned to a normal distribution as was also observed in PBS injected negative controls. In the case of N2306, which unlike the N843_10 isolate has an intact *prfA* gene, both the WT and *ΔrnjA* strains were not cleared by the immune system and were able to spread from the infection site, with the WT strain showing a higher spread intensity (Figure 10). The WT strain N2306 caused 100% zebrafish mortality at 24 hpi whereas N2306 Δ*rnjA* had only caused 10% mortality, and only reached 100% at 72 hpi (Supplementary Figure S9).

## Discusion

We examined in this study two *L. monocytogenes* isolates that were recovered within five years of each other from two episodes of a recurrent human PJI. Genomic analyses showed that the two *L. monocytogenes* isolates; N843_10 and N843_15 isolated in 2010 and 2015, respectively, were the same strain. Although displaying various minor genetic changes the two isolates clustered indistinguishably in a WGS-based phylogenetic tree and had less than 10 cgMLST allelic differences, which is currently considered a cut off for clonality among listerial strains (Ruppitsch et al., 2015). These two isolates displayed distinctive phenotypic traits suggesting that the relapse infection isolate N843_15 was an evolved variant of the original infection strain N843_10, which had evolved during the 5 years of residence within the human hip PJI environment. Besides impaired growth behavior and cellular morphology, the relapse infection isolate N843_15 also showed reduced motility, virulence and stress resistance as well as decreased carbon source utilization range, low biofilm production and altered antibiotic susceptibility compared to the N843_10 isolate. Comparing the genomes between the two strains revealed several genetic changes in the relapse infection isolate N843_15 including mutations in specific genes that might be linked to some of the phenotypic variations exhibited between this isolate and the original infection parent isolate N843_10. Presumably, these genetic changes arose from random mutation events that might be related to the evolution and adaptation of *L. monocytogenes* N843 strain to the niche of the infected human prosthetic joint environment. Others have also previously described phenotypically distinguishable clonally identical *L. monocytogenes* strains differing in phenotypic traits such as hemolysis, but these were isolated simultaneously from the same septic joint fluid (Charlier et al., 2012).

One of the genetic mutations in the evolved variant N843_15 was a single base deletion (106A) in the *rnjA* gene causing truncation of RNase J1(Table 5). RNase J1 is an RNA nuclease that plays an important role in bacterial RNA processing and degradation (Even et al., 2005; de la Sierra-Gallay et al., 2008; Figaro et al., 2013). The altered cellular morphology phenotypic defects observed in N843_15 such as the disordered peptidoglycan and cell chaining mirrors those previously described in *Bacillus subtilis* RNase J1 knock-down cells (Hunt et al., 2006; Mader et al., 2008; Durand et al., 2012; Figaro et al., 2013). Furthermore, we could recapitulate some phenotypic defects exhibited by N843_15 such as cellular filamentation as well as reductions in motility, antibiotic resistance and virulence through *rnjA* gene deletion in *L. monocytogenes* N843_10 and N2306 strains, which represent genetic backgrounds of the original PJI isolate as well as a different *L. monocytogenes* genetic lineage, respectively. The N843_10 and N2306 Δ*rnjA* mutant phenotypes were however less pronounced than those of the N843_15 isolate, which was presumably because the N843_15 phenotypic defects are probably exacerbated due to contributions of other mutations besides the RNase J1 truncation (Table 5). Possible contributing mutations include a single base insertion (769A) induced truncation of the D-alanine carboxypeptidase (PBDB2) protein Lmo2812 previously found to be important in peptidoglycan synthesis and cell wall turnover events in *L. monocytogenes* (Principe et al., 2009).

Reduction of flagella-based swarming motility observed in N843_15 could be due to altered cellular morphology since incompletely separated chained cells formed by this strain might be too large to be moved through flagella. Besides that, the peptidoglycan structural disordering observed in this isolate could also impair flagella assembly. Although it remains to be investigated, the flagellum specific muraminidase FlgJ in N843_15 also has a SNP induced V15C amino acid change, which might have possible functional consequences that could compromise flagella assembly and motility processes. As previously suggested by others, *L. monocytogenes* might also strategically down regulate the expression of motility related genes as a way of avoiding the activation of the host immune system, reduced motility in N843_15 might thus also be a deliberate adaptive change to long-term residence within the human host PJI environment (Toledo-Arana et al., 2009).

There was loss in the ability to utilize some carbon sources and increased sensitivity to both acidic and osmotic stress detected in the relapse isolate N843_15 compared to the parent isolate N843_10. Notably such losses included the ability to utilize intracellular and food relevant C-sources such as glycerol and pectin, respectively, as well as increased sensitivity to sodium chloride, sodium lactate, and sodium benzoate stresses. It is tempting to speculate that sacrificing of such phenotypes might be related to the evolution of the *L. monocytogenes* N843 strain as it adapted to the human host PJ capsule environment. At genome level, the changes in C-source utilization ability in N843_15 might in part be associated with amino acid changing and truncation inducing mutations of carbon metabolism related proteins such as the maltose phosphorylase (loss of D-maltose utilization ability) and galactitol operon BglG anti-terminator, BvrA. Meanwhile increased osmotic and acid stress sensitivities could have arisen from the peptidoglycan structural alteration, as well as ClpQ, HtrA and ArcD truncation mutations found in the N843_15 genome. Proper peptidoglycan architecture is important for bacterial survival in diverse environments and as previously shown in *Lactobacillus* and *Ochrobactrum* specie*s* its alteration increases osmotic and acid stress sensitivity (Piuri et al., 2005; Principe et al., 2009; Alonzo et al., 2011; Bergholz et al., 2012; Vollmer, 2015). Proteases HtrA and ClpQ promote general stress responses through degradation of misfolded proteins, whereas ArcD promotes acid stress tolerance via the ADI system by facilitating the exchange of intracellular ornithine for extracellular arginine in *L. monocytogenes* (Stack et al., 2005; Bowman et al., 2008; Kocaman and Sarımehmetoǧlu, 2016). In addition, proteins with DD carboxypeptidase activity are also involved in cell wall turnover as they function to cleave peptidoglycan cross-links (Piuri et al., 2005; Korsak et al., 2010). Similar with previous observations for DD-carboxypeptidase mutants in *Ochrobactrum* specie*s* and *L. monocytogenes*, the N843_15 isolate that also bears a D-alanine carboxypeptidase (PBDB2-*lmo2812*) truncation mutation also displayed increased sensitivity to osmotic stress compared to the parent isolate N843_10 (Principe et al., 2009; Bergholz et al., 2012). Although not yet proven, the overall reduction in ability to utilize intracellular carbon sources, as well as low acid stress tolerance and hemolytic ability might be reflective of N843_15 adaptation to an extracellular life within the human prosthetic joint environment.

N843_10 and N843_15 isolates were both clinically sensitive based on Clinical and Laboratory Standards Institute and European Committee on Antimicrobial Susceptibility Testing standards against antibiotics tested including those associated with the PJI therapy (CLSI, 2010; European Committee on Antimicrobial Susceptibility Testing, 2017). This observation rules out the possibility that the evolved mutant isolate N843_15 had emerged through increased antibiotic resistance and subsequent selection during PJI treatment. Both strains could, however, have survived due to antibiotic therapy failure since bactericidal antibiotic levels might not have been achieved in the PJ environment during therapy. On the other hand, N843_15 was more sensitive to most of the tested antibiotics than the parent isolate N843_10 probably due to increased antibiotic permeability due to its peptidoglycan structural alteration or as a result of its various specific acquired mutations. A 50S ribosomal protein L15 subunit truncation mutation found in this isolate might explain the increased sensitivity to the 50S targeting antibiotics tetracycline and azithromycin. The HtrA and RNase J1 truncation mutations could increase sensitivity to penicillin and other antibiotics in N843_15 since previous studies showed loss of these proteins increases antibiotic sensitivity in *L. monocytogenes* and *B. subtilis*, respectively (Stack et al., 2005; Figaro et al., 2013). The contribution of RNase J1 loss to increased antibiotic sensitivity was also corroborated since both N843_10 and N2306 Δ*rnjA* mutants also showed increased antibiotic sensitivity. Similar with observations upon RNase J1 depletion in *B. subtilis*, *L. monocytogenes* N843_15 was also more resistant to the dihydropteroate synthetase inhibitor sulfamethoxazole than the parent N843_10 isolate (Hunt et al., 2006). Increased sulfamethoxazole resistance was also observed for N843_10 *ΔrnjA* but not the N2306 Δ*rnjA* mutant, which indicates some genetic lineage and strain-specific differences regarding the contribution of RNase J1 loss to this phenotype in *L. monocytogenes*. Similarly, strain specific variation in sensitivity phenotypes has also been described among RNase J1 mutants created in different *B. subtilis* strains against the dihydrofolate reductase inhibitor trimethoprim, which like sulfamethoxazole also targets a stage in the folate pathway (Hunt et al., 2006; Figaro et al., 2013).

Prosthetic joint infections pathogenesis varies from that of a native joint since it relies on biofilm formation and *L. monocytogenes* has a high affinity for implants (Francolini and Donelli, 2010; Bader et al., 2016). Biofilm environments protect the bacterium from exposure to antibiotics and possibly the immune system (Kleemann et al., 2009). Although it remains possible that the relapse isolate N843_15 could also have persisted as a biofilm in the PJI our analysis here showed that it produces significantly less biofilm than the parent isolate N843_10. A possible explanation for this could be the HtrA truncation mutation in this isolate since reduced biofilm production has been described in *htrA* null mutants of *L. monocytogenes* (Wilson et al., 2006). We, however, deem it unlikely that N843_15 survived overtime in the PJI due to an increased biofilm production ability compared to the original N843_10 isolate, although it is important to note that the biofilm comparison was conducted under laboratory conditions using attachment surfaces that differ from those encountered within the human prosthetic joint.

Virulence analysis showed that the relapse infection isolate N843_15 has reduced cell invasion capacity than the original infection isolate N843_10, which might probably be associated with altered display and anchoring of cell surface associated virulence factors including internalins due to the filamentous morphology and disordered peptidoglycan in this strain. *L. monocytogenes* virulence depends on a variety of virulence factors that are transcriptionally regulated through PrfA (Chatterjee et al., 2006; Desvaux and Hebraud, 2006; Cossart and Lebreton, 2014; Radoshevich and Cossart, 2018). One hypothesis is that both N843 isolates might have persisted in the prosthetic joint due to low virulence that limited deleterious effects to the host. In support of this notion both N843 isolates displayed low virulence than other reference *L. monocytogenes* strains tested based on hemolysis, cell invasion and zebrafish pathogenicity assessments. The low virulence in the N843 isolates was associated with a *prfA* truncation mutation similar to previous observations in other *L. monocytogenes* strains (Maury et al., 2017). In both N843 PJI isolates this was due to a *prfA* deletion mutation that truncates the PrfA protein within the dimerization domain. Site directed mutagenesis of amino acid residues within this PrfA region was previously shown to impair DNA binding and virulence gene expression causing low virulence and LLO secretion (Eiting et al., 2005; Scortti et al., 2007; de las Heras et al., 2011; Good et al., 2016). Meanwhile the slightly elevated *prfA* mRNA levels detected in N843_10 and N843_15 compared to a control CC412 strain N12_1273 might be indicative of increased production of truncated PrfA to compensate for reduced activity. Such increased expression might be driven through other transcriptional regulators such as alternative sigma factor Sig B (Lebreton and Cossart, 2017). Interestingly, *prfA* mRNA levels were also higher in N843_15 compared to N843_10. We, however, do not have an explanation for this, but it might be linked to other mutations that N843_15 has in addition to the PrfA truncation.

In a simulation of localized infection using different zebrafish lines, isolate N843_15 and the Δ*rnjA* mutant of N843_10 were not cleared by the immune system over 48 hrs and neither did they cause any overt disease signs. Notably although macrophages were initially attracted to the bacterial injection site, they returned to normal distribution without clearing cells of these strains from the infection site. Such a response pattern was also observed for the PBS injected negative controls. Although mechanisms behind this phenotype are not yet clear, one possible explanation might the altered expression of motility and virulence associated genes. Furthermore, the detected mutations might have an impact on the expression and secretion of Pathogen-associated molecular patterns (PAMPs) such as flagellin and LLO, thereby inducing a different response from the immune system compared to other normal wild type *L. monocytogenes* strains (Rose et al., 2001; Way et al., 2004; Schuppler and Loessner, 2010; Wallecha et al., 2013).

A genome comparison between N843_10 and N843_15 revealed various genetic variations some of which might explain the phenotypic differences detected between the two N843 isolates. It is, however, important to note that going forward specific gene targeting mutagenesis and complementation approaches will be necessary to validate some of the observations reported here. Meanwhile, the N843_15 genome changes detected relative to the N843_10 parent strain demonstrates vestigiality suggestive of short-term adaptation through inactivation and retainment of the unnecessary genes. In long-term adaptation, an eventual complete loss of the unnecessary genes would be expected. In contrast to findings by others, our study shows relatively higher level of genetic variability between the two strains which differs from high level genome conservation observed from long-term evolution of *L. monocytogenes* in other settings such as food processing environments (Orsi et al., 2008; Lomonaco et al., 2015; Moura et al., 2016; Harranda et al., 2020). A likely contributor to the relatively high mutation rate could be that the strains were replicating at a relatively higher rate within the PJI environment at 37°C, than they would in food production plant environments upon which previous observations are based (Orsi et al., 2008; Lomonaco et al., 2015; Harranda et al., 2020). We are, however, aware that such a comparison of mutation rates might be confounded due to differences in strain population sizes and genetic backgrounds as well as environmental niches involved between these other studies and our current study that only involved two isolates within the PJI environment.

In conclusion, we have characterized two clonally identical *L. monocytogenes* strains N843_10 and N843_15 that were isolated 5 years apart from a persistent rare manifestation of listeriosis. We show that the long-term residence of this bacterium within a human host PJI environment led to a wide range of phenotypic changes in virulence, metabolic flexibility, and stress resilience that were associated with various genetic changes during short-term evolution of *L. monocytogenes* within a human PJI environment. Overall our observations besides highlighting the phenotypic and genotypic variations between these two strains might have provided some insights into molecular mechanisms associated with adaptation and evolution of *L. monocytogenes* within the environment of a human host PJI. It is, however, important to note that our case might be a special case involving strains that carry a natural truncation mutation in the main virulence regulator PrfA. As such, more case studies would be required in future in order to generalize our findings regarding mechanism of adaptation of other *L. monocytogenes* strains during persistence within the human host PJI environment.

## Data Availability Statement

The datasets generated for this study can be found in the NCBI GeneBank – Accession numbers CP046361 and CP046362.

## Ethics Statement

This study was performed in accordance with the principles and recommendations of the “Ordinance on laboratory animal husbandry, the production of genetically modified animals and the methods of animal experimentation; Animal Experimentation Ordinance” (SR 455.163, April 12, 2010), Swiss Federal Food Safety and Veterinary Office (FSVO/BLV). The maximum age reached by the embryos during experimentation was 5 days post fertilization (dpf) for which no license is required from the cantonal veterinary office in Switzerland, since such embryos will not have yet reached the free-feeding stage. Husbandry and breeding of the adult zebrafish were performed under the supervision of Prof. Stephan Neuhauss, Institute for Molecular Life Sciences, University of Zurich, Zurich, Switzerland. All animal protocols used were in compliance with internationally recognized standards as well as with Swiss legal ethical guidelines for the use of fish in biomedical research. All the experiments were approved by the local authorities (Veterinäramt Zürich Tierhaltungsnummer 150).

## Author Contributions

TT and FM designed the study. TT supervised the study. FM and AE performed the experiments. UA assisted in the PM experiments. FM, AE, UA, MS, and TT analyzed the data and wrote the manuscript. All authors contributed to the article and approved the submitted version.

## Conflict of Interest

The authors declare that the research was conducted in the absence of any commercial or financial relationships that could be construed as a potential conflict of interest.

## Footnotes

1 http://bigsdb.web.pasteur.fr/listeria/listeria.html

2 https://biolog.com

3 https://rast.nmpdr.org/

4 https://bigsdb.pasteur.fr/listeria/

5 https://www.cgmlst.org/ncs/schema/690488/
