# Supplementary material for: Evolution of Listeria monocytogenes During a Persistent Human Prosthetic Hip Joint Infection
Source: Front Microbiol. 2020 Jul 28;11:1726. doi: 10.3389/fmicb.2020.01726 (PMC7399150; doi:10.3389/fmicb.2020.01726)
Supplement: Supplementary file 1 [file Table_1.docx]

**Supplementary Table S1. Primers used in this study**

| **Gene target** | **Primer sequence (5′–3′)** | **Protein** | **Purpose** | **Reference** |
| --- | --- | --- | --- | --- |
| Δ*rnjA* | Fwd—GTTGGAGTAAGAGGCATT  Rev—GTAGAACGACAATGGAAC | RNase J1 mutant creation check | Δ*rnjA* mutant selection | This study |
| *rnjA* | Fwd—TTTGTTGCGGATAAGTGC  Rev—CACTATATGTAGAAGCGG | RNase J1 | Mutation confirmation | This study |
| *htrA* | Fwd—CACAGACCCTGATAATGCTAC  Rev— ACCACTTCCGCCATTATTTAC | HtrA | Mutation confirmation | This study |
| *lmo2812* | Fwd— CAACTTCCAAACCCACTGTC  Rev—TACTTCGGTCTGCTACTGC | Penicillin-binding proteins D2 | Mutation confirmation | This study |
| *lmo1799* | Fwd— TGAGAATAATCCAAGCCCAAC  Rev—GGAGATTAGACCAAAAGCGAG | Putative PBP (LPXTG motif) | Mutation confirmation | This study |
| *lmo0842* | Fwd— TGGTGTAGCAATTCCTTTCTC  Rev—TGGTCACCTGTTTTTGGTAATG | Putative peptidoglycan bound protein (LPXTG motif) | Mutation confirmation | This study |
| *16S rDNA* | Fwd—CTTCCGCAATGGACGAAAGT  Rev—CTCATCGTTTACGGCGTG | Small ribosomal RNA subunit | RT-qPCR | Eshwar et al., 2017 |
| *plcA* | Fwd—TCGGGGAAGTCCATGA  Rev—GGCGCACCTAACCAAG | Phosphatidylinositol phospholipase C (PI-PLC) | RT-qPCR | Eshwar et al., 2017 |
| *hly* | Fwd—ACCTCGGAGACTTACG  Rev—TCCTCCAGAGTGATCG | Listeriolysin O (LLO) | RT-qPCR | Eshwar et al., 2017 |
| *prfA* | Fwd—GGCTCTATTTGCGGTCAACT  Rev—GCTAACAGCTGAGCTATGTGC | Positive regulatory factor A (PrfA) | RT-qPCR | Maury et al., 2017 |

**Supplementary Table S4. MICs of different antibiotics determined for the *L. monocytogenes* RNase J1 mutants in comparison to their parent strains using E-tests**

| **Antibiotic^*^** | **N843-10 (μg/ml)** | **N843-10 ∆*rnjA* (μg/ml)** | **N2306 (μg/ml)** | **N2306 ∆*rnjA* (μg/ml)** |
| --- | --- | --- | --- | --- |
| Gentamicin**^1^** | 0.094 ± 0 | 0.094 ± 0 | 0.315 ± 0 | 0.06 4 ± 0 |
| Sulfamethoxazol | 12 ± 0 | 18 ± 0 | 12± 0 | 0.064 ± 0 |
| Amoxicillin | 0.5633 ± 0.15 | 0.42 ± 0.07 | 0.094 ± 0 | 0.023 ± 0 |
| Cephalothin | 4.5 ± 1 | 4 ± 0 | 2.5 ± 0.58 | 0.5 ± 0 |
| Vancomycin | 1.5 ± 0 | 1.5 ± 0 | 1.8333 ± 0.29 | 1.333 ± 0.29 |
| Rifampicin | 0.08 ± 0 | 0.08 ± 0 | 0.094 ± 0 | 0.064 ± 0 |

*Listed are antibiotics that *rnjA* deletion might have an impact on. **^1^**Gentamicin was included because it is a commonly used drug in listeriosis treatment. Presented are means and their standard deviation from 3 independent experiments.
