# Supplementary material for: Evolution of Listeria monocytogenes During a Persistent Human Prosthetic Hip Joint Infection
Source: Front Microbiol. 2020 Jul 28;11:1726. doi: 10.3389/fmicb.2020.01726 (PMC7399150; doi:10.3389/fmicb.2020.01726)
Supplement: Supplementary file 4 [file Presentation_1.pptx]

## Slide 1
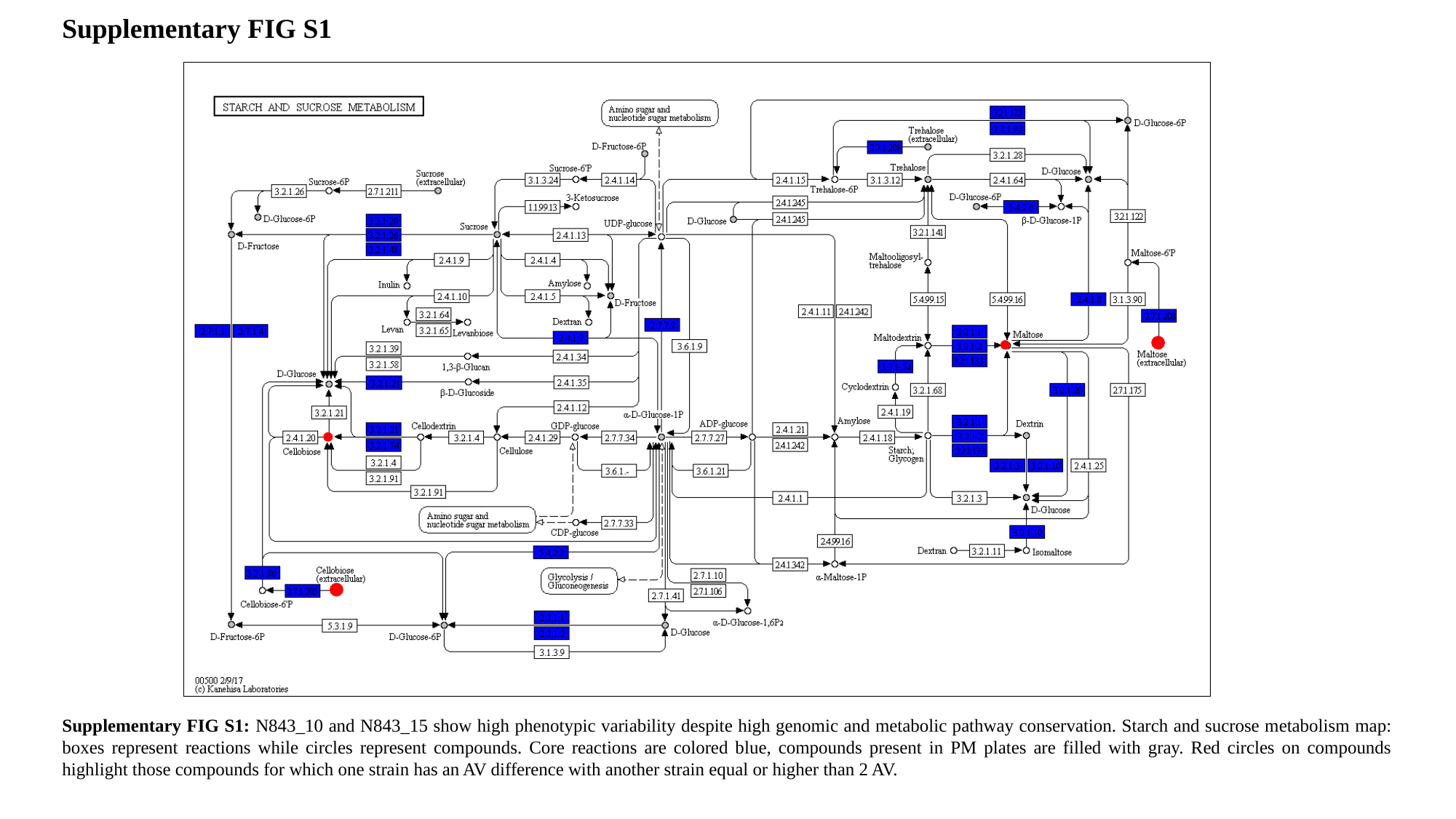

Supplementary FIG S1
Supplementary FIG S1: N843_10 and N843_15 show high phenotypic variability despite high genomic and metabolic pathway conservation. Starch and sucrose metabolism map: boxes represent reactions while circles represent compounds. Core reactions are colored blue, compounds present in PM plates are filled with gray. Red circles on compounds highlight those compounds for which one strain has an AV difference with another strain equal or higher than 2 AV.

## Slide 2
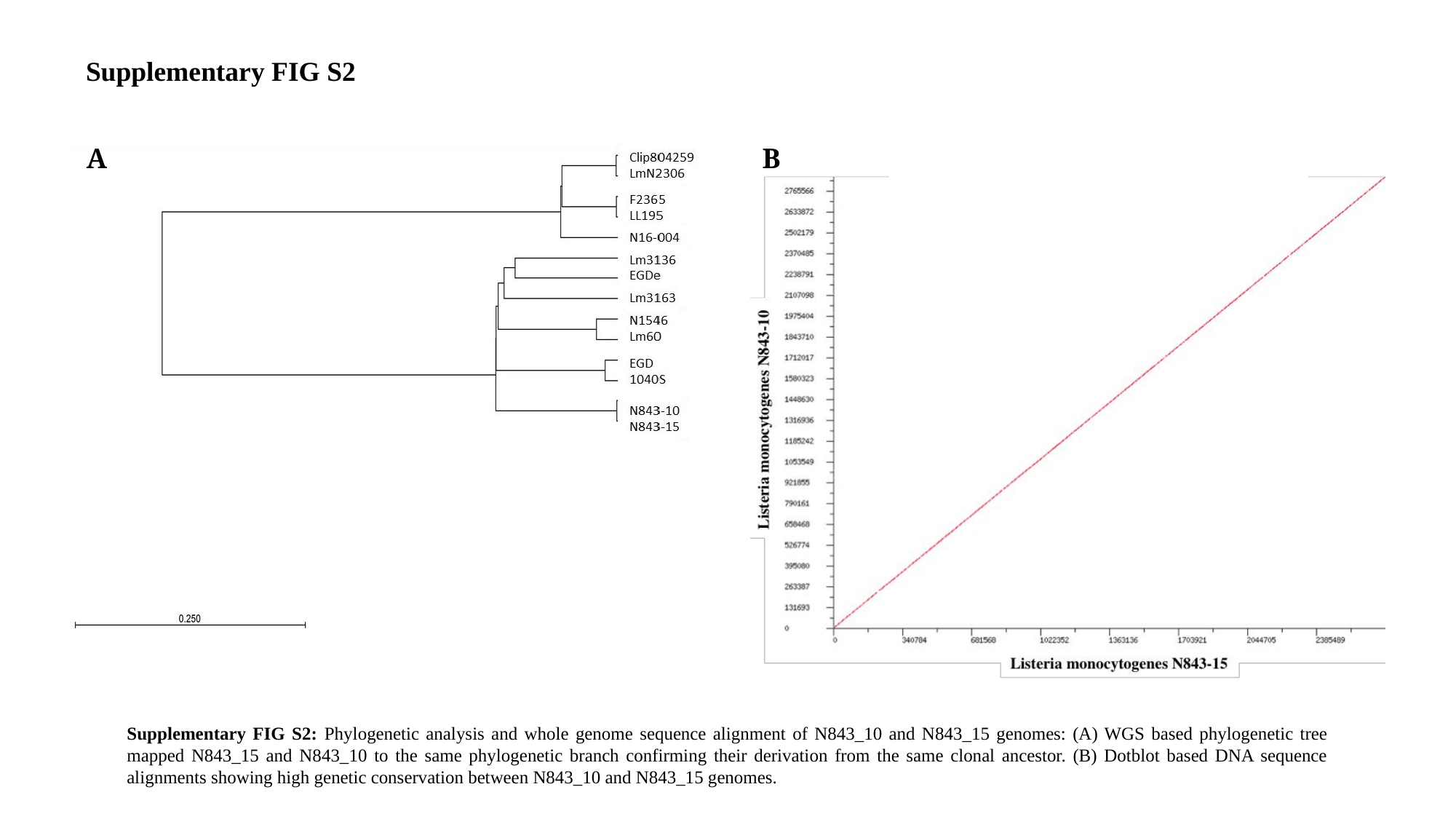

Supplementary FIG S2
A
B
Supplementary FIG S2: Phylogenetic analysis and whole genome sequence alignment of N843_10 and N843_15 genomes: (A) WGS based phylogenetic tree mapped N843_15 and N843_10 to the same phylogenetic branch confirming their derivation from the same clonal ancestor. (B) Dotblot based DNA sequence alignments showing high genetic conservation between N843_10 and N843_15 genomes.

## Slide 3
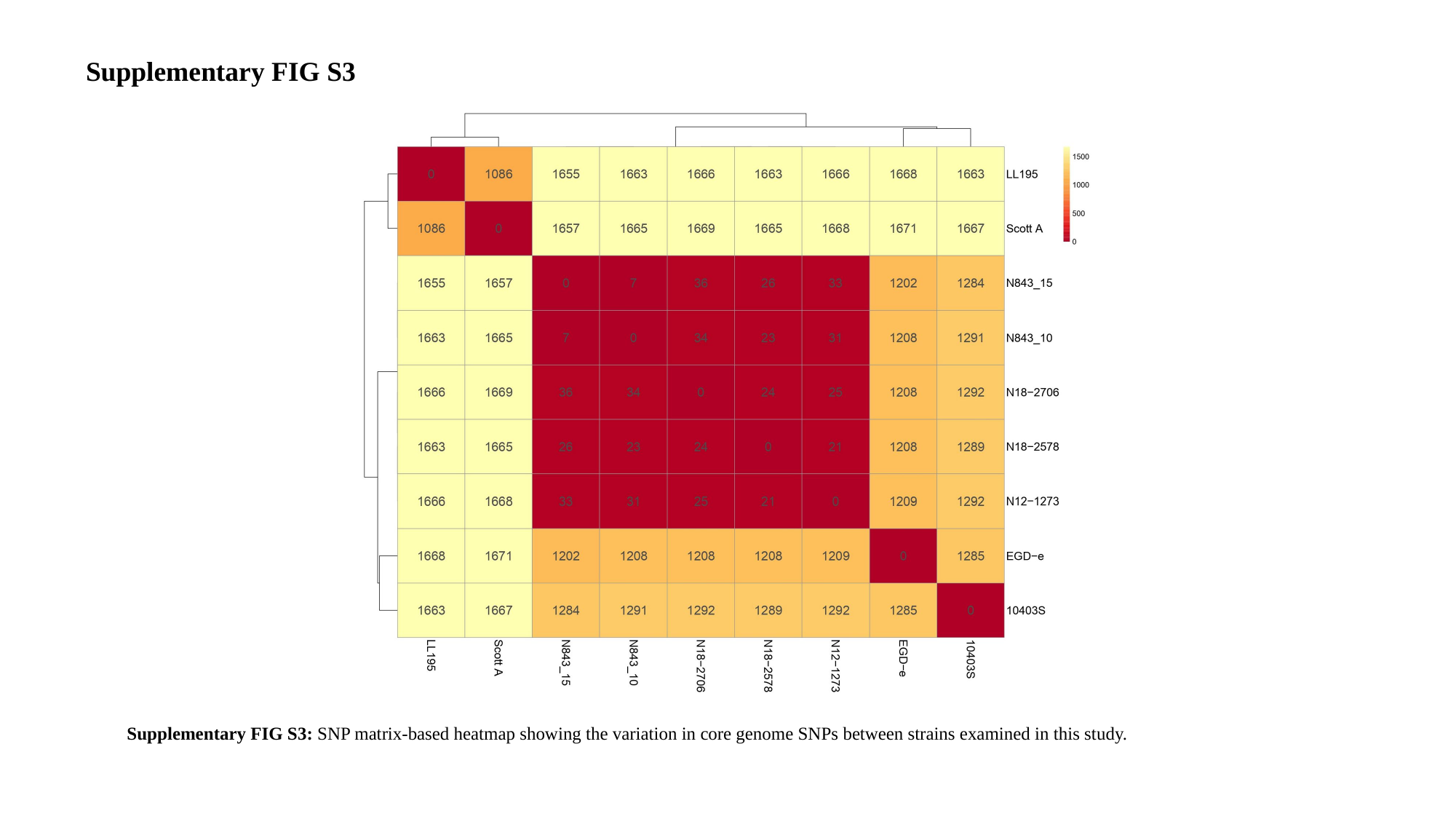

Supplementary FIG S3
Supplementary FIG S3: SNP matrix-based heatmap showing the variation in core genome SNPs between strains examined in this study.

## Slide 4
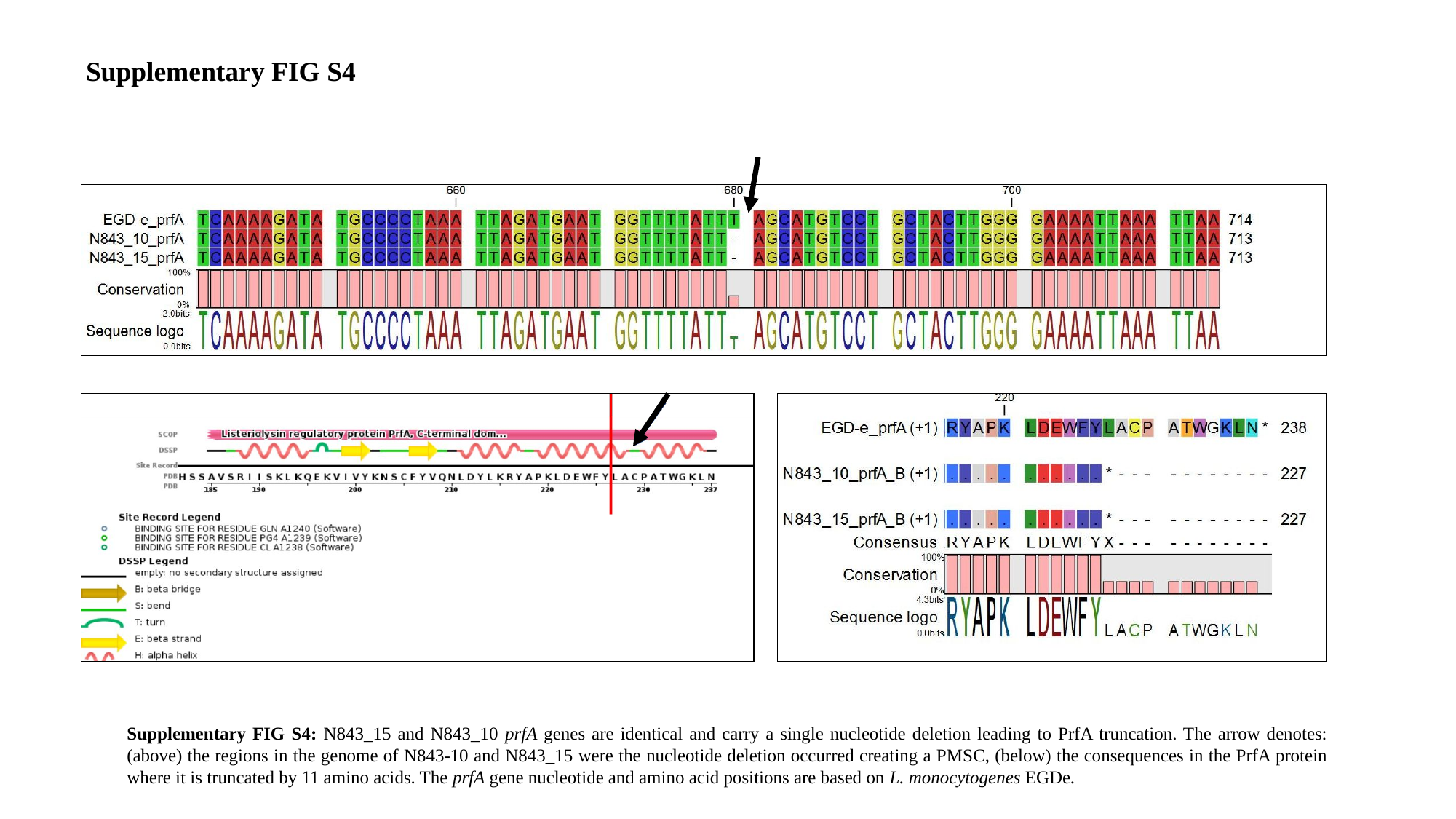

Supplementary FIG S4
Supplementary FIG S4: N843_15 and N843_10 prfA genes are identical and carry a single nucleotide deletion leading to PrfA truncation. The arrow denotes: (above) the regions in the genome of N843-10 and N843_15 were the nucleotide deletion occurred creating a PMSC, (below) the consequences in the PrfA protein where it is truncated by 11 amino acids. The prfA gene nucleotide and amino acid positions are based on L. monocytogenes EGDe.

## Slide 5
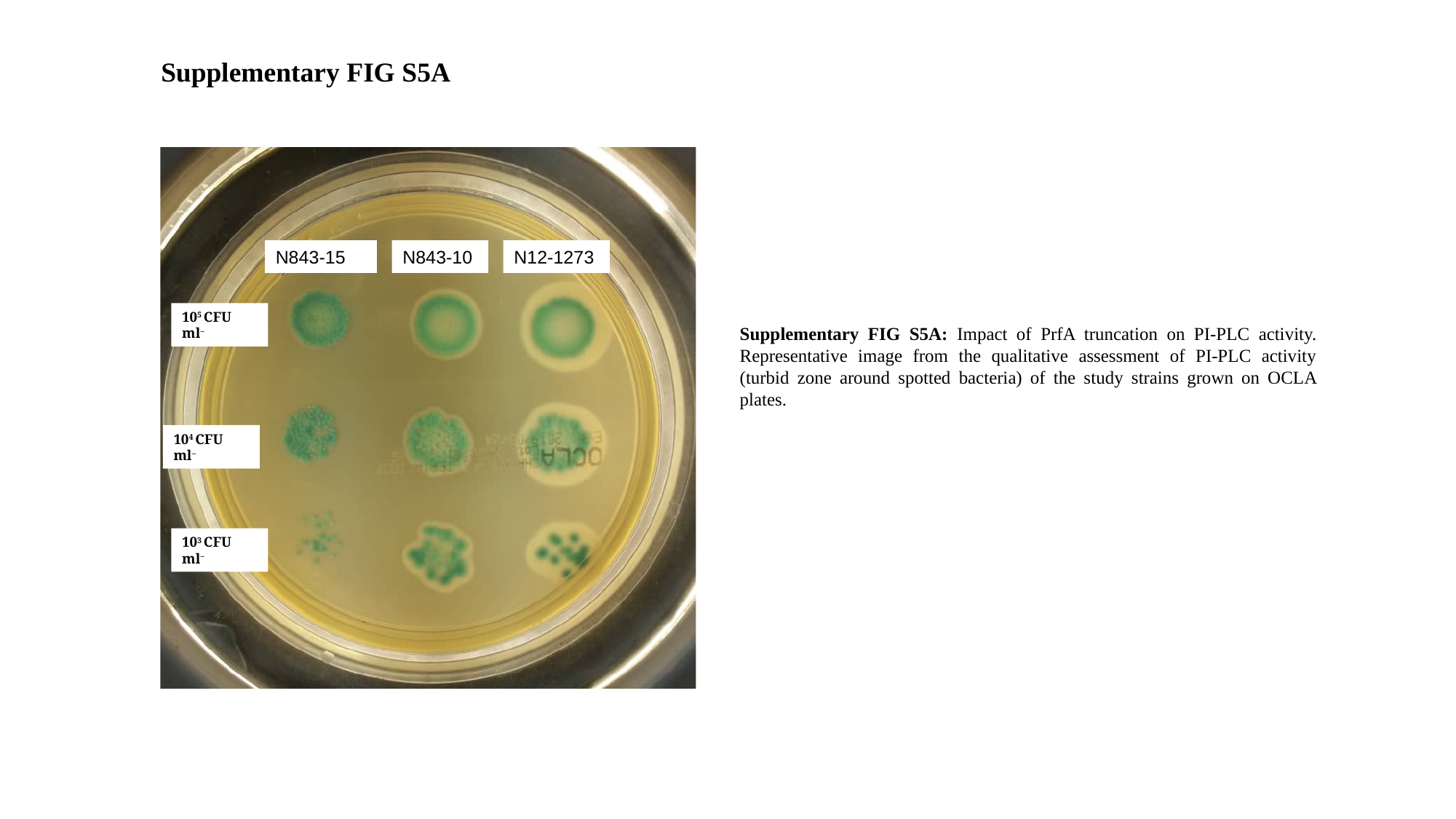

Supplementary FIG S5A
N843-15
N843-10
N12-1273
105 CFU ml−
104 CFU ml−
103 CFU ml−
Supplementary FIG S5A: Impact of PrfA truncation on PI-PLC activity. Representative image from the qualitative assessment of PI-PLC activity (turbid zone around spotted bacteria) of the study strains grown on OCLA plates.

## Slide 6
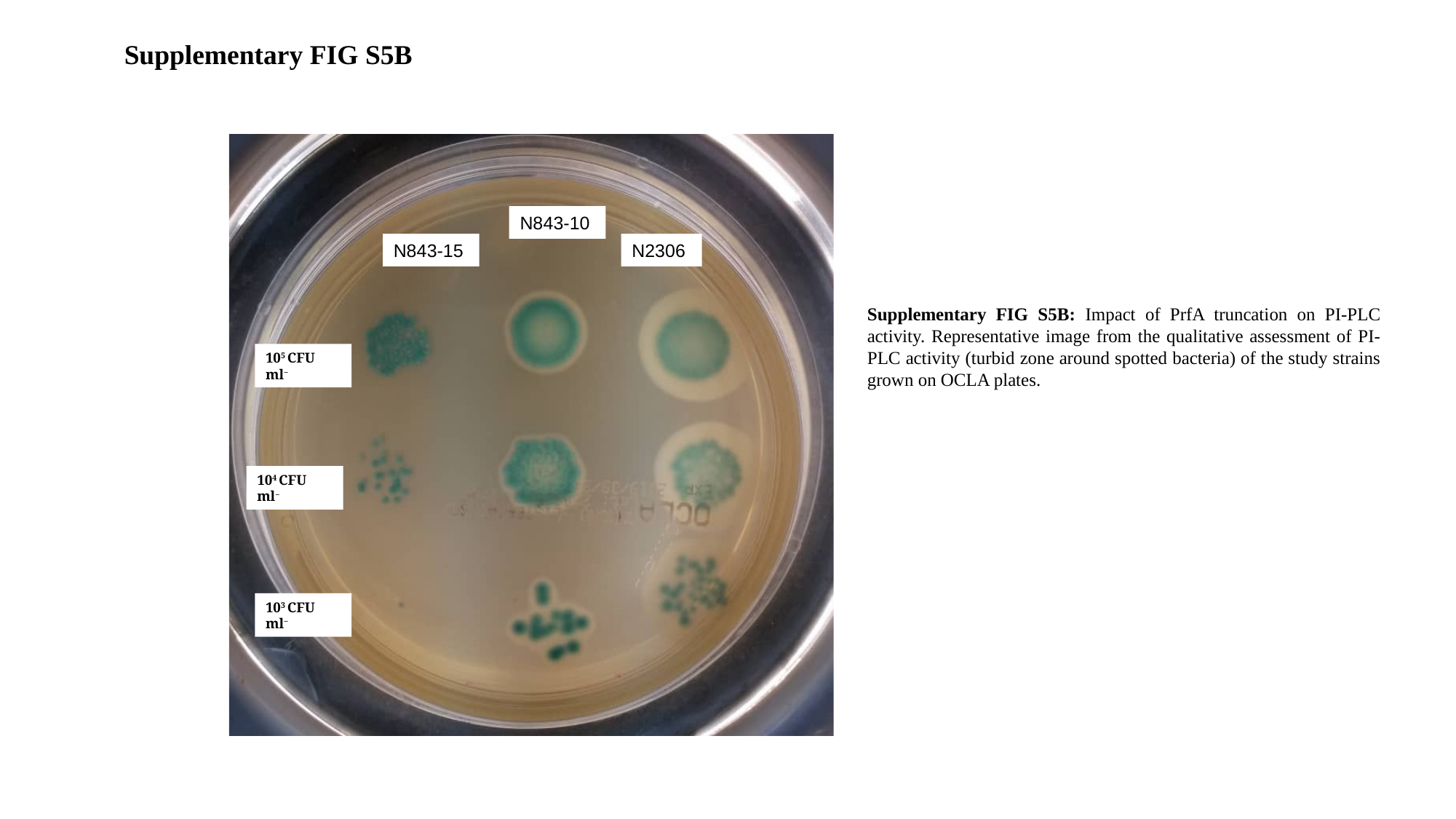

Supplementary FIG S5B
N843-10
N843-15
N2306
105 CFU ml−
104 CFU ml−
103 CFU ml−
Supplementary FIG S5B: Impact of PrfA truncation on PI-PLC activity. Representative image from the qualitative assessment of PI-PLC activity (turbid zone around spotted bacteria) of the study strains grown on OCLA plates.

## Slide 7
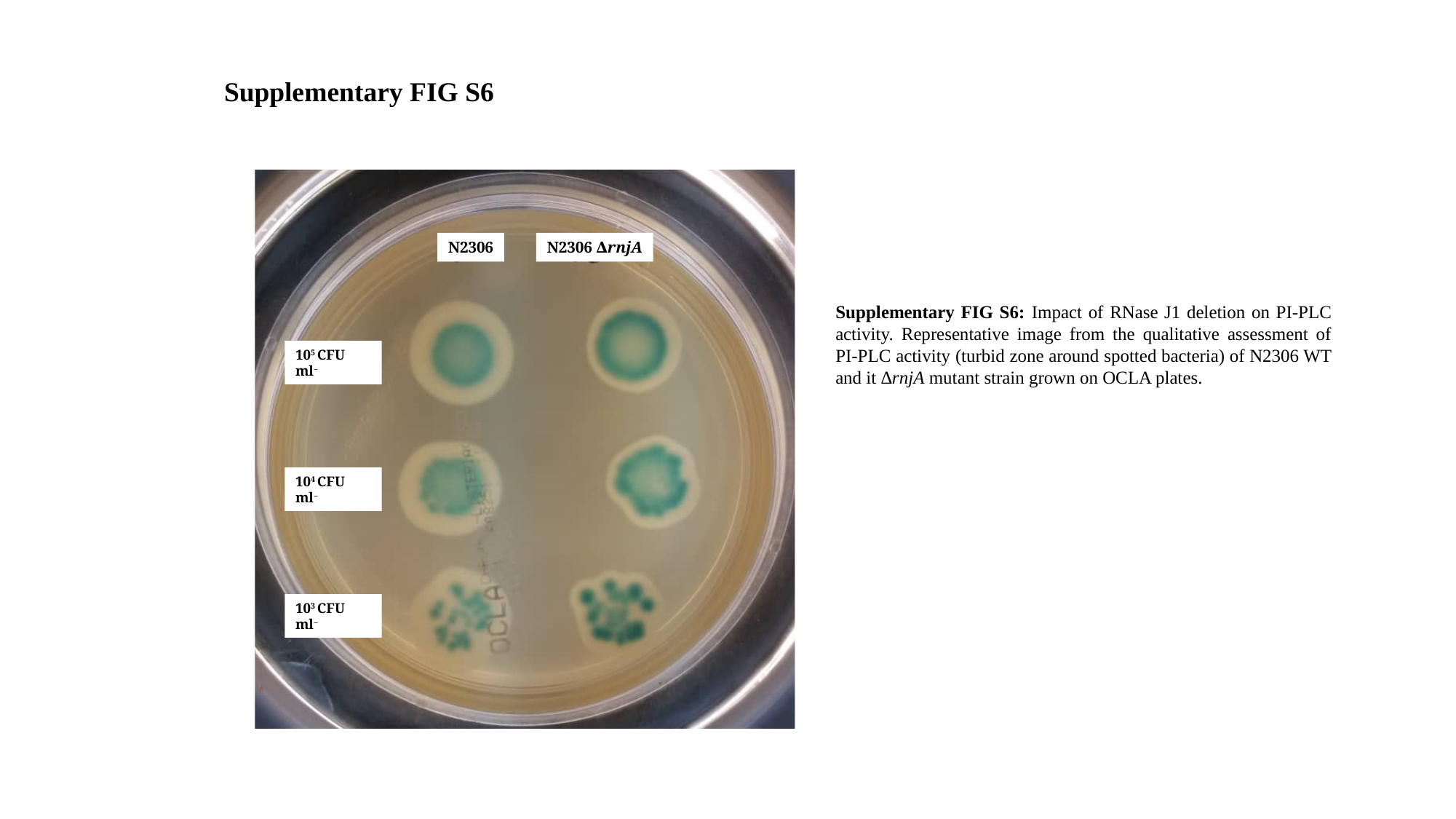

Supplementary FIG S6
N2306
N2306 ∆rnjA
105 CFU ml−
104 CFU ml−
103 CFU ml−
Supplementary FIG S6: Impact of RNase J1 deletion on PI-PLC activity. Representative image from the qualitative assessment of PI-PLC activity (turbid zone around spotted bacteria) of N2306 WT and it ∆rnjA mutant strain grown on OCLA plates.

## Slide 8
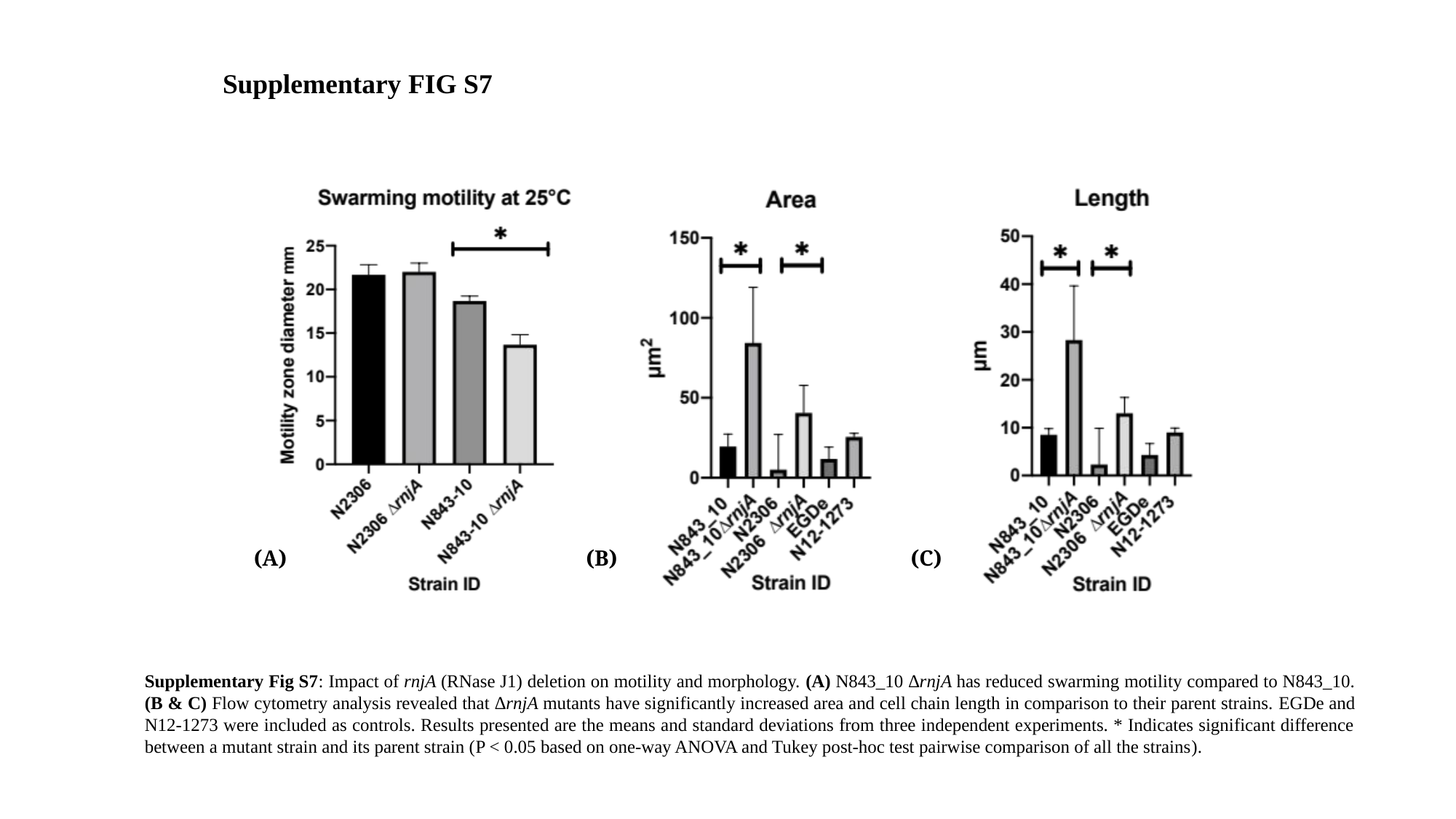

Supplementary FIG S7
(A)
(B)
(C)
Supplementary Fig S7: Impact of rnjA (RNase J1) deletion on motility and morphology. (A) N843_10 ∆rnjA has reduced swarming motility compared to N843_10. (B & C) Flow cytometry analysis revealed that ∆rnjA mutants have significantly increased area and cell chain length in comparison to their parent strains. EGDe and N12-1273 were included as controls. Results presented are the means and standard deviations from three independent experiments. * Indicates significant difference between a mutant strain and its parent strain (P < 0.05 based on one-way ANOVA and Tukey post-hoc test pairwise comparison of all the strains).

## Slide 9
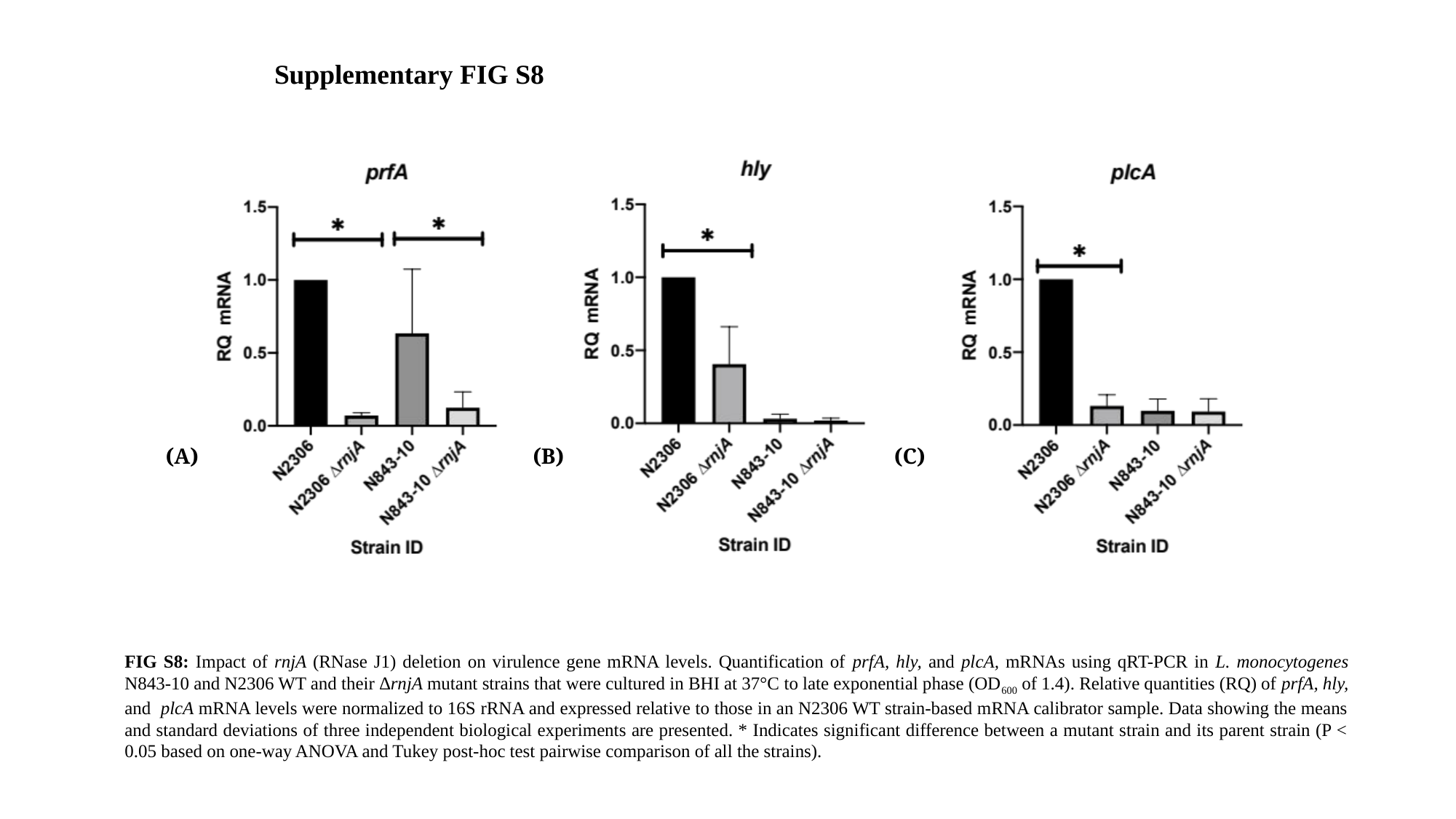

Supplementary FIG S8
(A)
(B)
(C)
FIG S8: Impact of rnjA (RNase J1) deletion on virulence gene mRNA levels. Quantification of prfA, hly, and plcA, mRNAs using qRT-PCR in L. monocytogenes N843-10 and N2306 WT and their ∆rnjA mutant strains that were cultured in BHI at 37°C to late exponential phase (OD600 of 1.4). Relative quantities (RQ) of prfA, hly, and plcA mRNA levels were normalized to 16S rRNA and expressed relative to those in an N2306 WT strain-based mRNA calibrator sample. Data showing the means and standard deviations of three independent biological experiments are presented. * Indicates significant difference between a mutant strain and its parent strain (P < 0.05 based on one-way ANOVA and Tukey post-hoc test pairwise comparison of all the strains).

## Slide 10
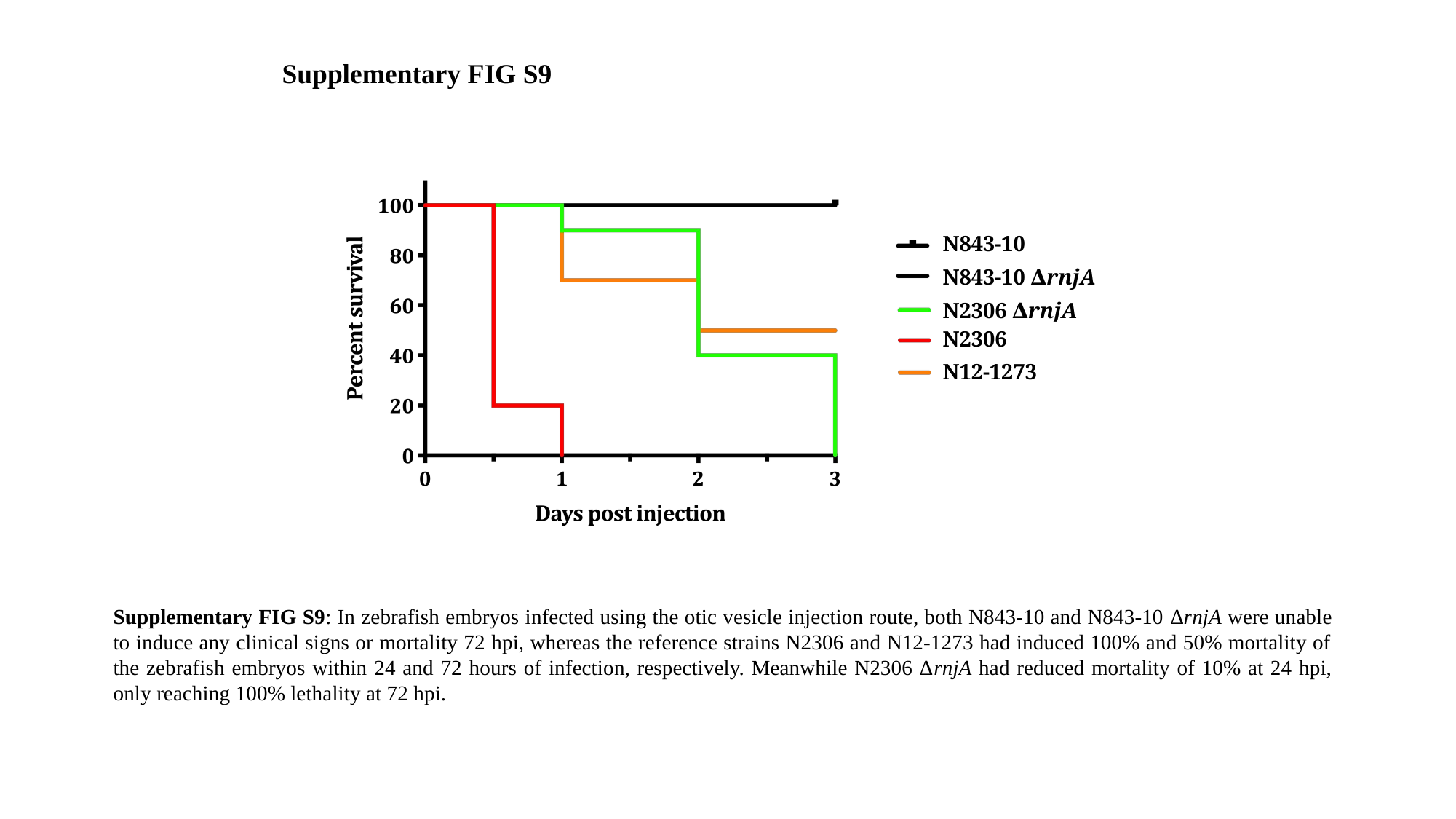

Supplementary FIG S9
N843-10
N843-10 ∆rnjA
N2306 ∆rnjA
N2306
N12-1273
Supplementary FIG S9: In zebrafish embryos infected using the otic vesicle injection route, both N843-10 and N843-10 ∆rnjA were unable to induce any clinical signs or mortality 72 hpi, whereas the reference strains N2306 and N12-1273 had induced 100% and 50% mortality of the zebrafish embryos within 24 and 72 hours of infection, respectively. Meanwhile N2306 ∆rnjA had reduced mortality of 10% at 24 hpi, only reaching 100% lethality at 72 hpi.
